# Effect of social and behavioral change interventions on minimum dietary diversity among pregnant women and associated socio-economic inequality in Rajasthan, India

**DOI:** 10.1186/s40795-024-00887-1

**Published:** 2024-06-06

**Authors:** Anshita Sharma, Srei Chanda, Akash Porwal, Namita Wadhwa, Divya Santhanam, Raghwesh Ranjan, Hemang Shah, Shachi Adyanthaya, Ramavatar Meena

**Affiliations:** 1 Social and Economic Empowerment, IPE Global Limited, IPE Global Limited, Delhi, India; 2 Child Health and Development, Children’s Investment Fund Foundation, Delhi, India; 3 Directorate of Integrated Child Development Services, Jaipur, Rajasthan India

**Keywords:** Minimum dietary diversity, Pregnancy Care, SBCC, Rajasthan

## Abstract

**Background:**

Maternal dietary diversity is a key to improving the birth and child health outcomes. Besides socio-economic factors, the nutrition specific program- Social and Behavioural Change Communication (SBCC) interventions aimed to improve maternal dietary diversity has varied levels of impact on the socio-economic groups in poor resource setups.

**Objective:**

To measure the factors associated with the minimum dietary diversity (MDD) among pregnant women in selected districts of Rajasthan with special emphasis on the SBCC components. Additionally, it measures the socio-economic gaps in the behaviour of consumption of diversified diet during pregnancy.

**Methods:**

Data from a cross sectional survey of 6848 pregnant women, who have received a continuous SBCC counselling and registered under a state introduced conditional cash transfer program, during May to June, 2023, in five intervention districts -Banswara, Baran, Dunagrpur, Pratapgarh and Udaipur in Rajasthan was used. A 24 h recall based food consumption behaviour has been gathered to measure the MDD of pregnant women. Study has used descriptive statistics, multivariate regressions, and multivariate decomposition analysis to address the research objectives.

**Results:**

Study finds that only 55.2% of pregnant women are consuming diverse diet in the study duration with mean dietary diversity score is 4.8 (+/- 1.5). Logistic regression finds that SBCC components such as frontline workers (aOR = 1.3, CI: 1.1–1.4), community motivators (aOR = 1.9, CI: 1.7–2.1), and participation in MCHND (aOR = 1.0, CI: 0.9-1.2) have significant and higher likelihood on consumption of MDD food on previous day. A higher education and belonging from richer wealth quintile also show higher association for consumption of MDD. Multivariate decomposition shows, among richest and poorest wealth categories there is 19% point difference (58% difference due to coefficient vs. 42% difference due to composition) in MDD consumption. This is positively contributed by the caste and educational categories of women.

**Conclusion:**

Despite a predominant vegetarian diet consuming population, better maternal dietary diversity was observed among those exposed to higher dose of SBCC intervention package. Educational status and caste of the respondent were significantly associated with minimum dietary diversity and contributed to the socio-economic inequality highlighting the importance of tailored and sustained SBCC interventions.

**Supplementary Information:**

The online version contains supplementary material available at 10.1186/s40795-024-00887-1.

## Background

Maternal dietary intake is a critical and modifiable risk factor determining child health outcomes and mortality [1]. India shares a significant burden of neonatal mortality (NM) as it contributes 60% of all neonatal deaths in South Asian countries (0.9 million) in 2017 [2]. Maternal malnourishment leading to inadequate weight gain during gestation results in adverse birth outcomes such as preterm birth, short for gestational ages and contributing significantly to NM [3–5]. Globally, 20 million low birth weight (LBW) children are born, and India shares 40% of the burden. Additionally, one-third of the low birth weight (LBW) babies are short for gestational age, while the rest are preterm [6]. To end all forms of malnourishment, Sustainable Development Goals aim to reduce the prevalence of LBW by 30% by the end of 2030. India has also launched the National Nutrition Mission/ POSHAN Abhiyaan with the aim of a 2-percentage points reduction in the prevalence of LBW annually between 2017 and 2022 [5].

Often, in low- and middle-income countries, women start pregnancy with low BMI, resulting in a high demand for a balanced energy and protein intake during pregnancy [7]. Consumption of 5 out of 10 food groups is required to maintain nutrient adequacy among pregnant women [8]. However, women in India consume fewer food groups than their household members, resulting in low dietary diversity [9, 10]. The dietary practices of pregnant women are determined by a complex nexus of biological, physiological, psychological, and socio-economic factors [11]. Gestational weight gain is highly correlated with the consumption of energy rich food, carbohydrate, monosaccharide and saccharose [12]. In the Asian sub-continent, the dietary consumption of cereals is much higher than other food groups [13, 14]. However, consumption of carbohydrates is often insufficient, which negatively affects the nutritional outcomes. In particular, overall dietary consumption of nutritious foods in the lower socio-economic strata was less diverse than in reference groups [15]. Despite its significance, only a few studies have been conducted in small geographies and communities to measure the effect of maternal dietary intake on birth outcomes in India ( [15–17] .

The fundamental approach identified to improve maternal nutrition comprises three major intervention processes - food fortification/ supplementation, cash transfers or incentives and behavioural change interventions. These follow a pathway to enhance calorie consumption and micronutrient intakes that, in turn, affect maternal health outcomes [18]. Together, nutrition-specific interventions such as counselling – behavioural change interventions and education/ awareness and nutrition-sensitive interventions – cash incentives have compounding effect on the maternal dietary diversity compared to a specific intervention [19]. However, pathways determining the change across population groups have been a contesting topic. Any positive change is observed as a trickledown effect from higher to lower socio-economic groups in each population group [20]. Further, it is found that the impact of the state-sponsored scheme(s) through intervention programs shows a divergent effect among the rich and poor, though the barriers faced by the targeted groups are yet to be determined in this context [21].

## Program interventions

Rajasthan, the largest state in India with a high burden of malnourishment, has shown a sluggish decline in neonatal mortality [2]. As per the latest round of National Family Health Survey of India (2019-21), the neonatal mortality rate in Rajasthan was reported as 20.2 per 1000 live births, along with 17% and 18% of children reported as having low birth weight and wasted, respectively. Therefore, it underscores the gravity of malnourishment among children and emphasizes the importance of addressing maternal malnutrition as the proximate determinant for the same.

RajPusht, launched in 2017 and implemented by IPE Global in five selected districts (Banswara, Baran, Dungarpur, Pratapgarh and Udaipur), is a program supported by Children’s Investment Fund Foundation (CIFF) - that works with various departments of Government of Rajasthan to accelerate the reduction in the prevalence of low birth weight and wasting by addressing the intergenerational effect of malnutrition. A multi-level SBCC strategy was deployed under the program, focusing on establishing a conducive environment by emphasizing the importance of maternal and child nutrition at the household and community level. As part of the intervention design, pregnant women were registered and given nutrition-specific interpersonal counselling by community volunteers (Poshan Champions) (see Table 1). Further, the capacity of frontline workers to deliver nutrition-sensitive counselling during pregnancy and early childhood at the household and community level was also developed by providing continuous mentoring and handholding support. An array of mid-media interventions was deployed to reiterate the essential messages at various forums so that there is a strong recall of such messages being delivered via interpersonal counselling (see Table 1). The SBCC strategy also targeted men and other family members, such as mothers-in-law, who were the prime decision-makers at the household level. Besides providing regular monitoring, the program conducted concurrent assessments of program quality biannually on several demographic, socio-economic, program intervention indicators and knowledge, attitude, and practice indicators. The program also supported the design and implementation of conditional cash transfer schemes in the intervention area. The primary objective of such cash transfer schemes was to supplement the households with additional resources to act on the dietary advice delivered through the SBCC interventions. In addition, the conditionalities of the schemes also act as a solid nudge to influence health-seeking behaviour within the target communities.

The present research argues for the effectiveness of nutrition-specific programs on the dietary diversity of pregnant women in the intervention districts. The study’s objective is to examine the effectiveness of various SBCC interventions deployed under RajPusht Program and measure the other socio-economic factors associated with the minimum dietary diversity of pregnant women. Secondly, to measure the rich and poor gaps in the behaviour of consuming diverse diets by pregnant women.

**Table 1:** Details of SBCC touch points under RajPusht program

Sr. No.	SBCC Intervention Mode	Details
1	**PC**	Poshan Champions (PCs) are the grass root level workers trained in Inter-Personal counselling, appointed under the RajPusht program. PCs register pregnant women and give them periodic home based inter-personal counselling on several vital issues - dietary practices, ANC care, vaccination, child feeding practices etc.
2	**MCHND**	Maternal, Child Health and Nutrition Day (MCHND) is conducted on Thursday and once in every month in a particular block. During session PW along with mother-in-law and husband were given counselling on dietary practices, pre and post pregnancy care and support.
4	**ANM**	Auxiliary Nurse and Midwife (ANM) provides health care support to the women and children. ANMs were trained to give nutrition specific counselling under the RajPusht.
5	**AWW**	Anganwadi Workers (AWWs) do home visits along with PCs to improve the counselling and influence the women with the knowledge and motivate them to improve the dietary practices during pregnancy. AWW were trained to give nutrition specific counselling under the RajPusht.

## Methods

### Data

For this study, data were used from the cross-sectional survey conducted biannually to monitor the progress in outcome indicators of the program. Data was collected by third-party field enumerators, who had been thoroughly trained on survey tools, probing, and handling sensitive questions. Data from the latest survey round conducted between May and June 2023 was used for analysis for this study. Data quality was ensured through field monitoring, backcheck, and spot check during the survey.

A total of 6848 pregnant women (respondents) were surveyed using a structured survey tool on a mobile-based application. The survey tool was field-tested and modified after a pilot study to monitor the coverage and effectiveness of the program precisely. It consisted of various questions to identify respondent’s socio-economic status and demographic details, including closed-ended questions on age, level of education, ownership of household assets, and household built-up characteristics. The survey tool also included closed-ended questions on the consumption of food items, knowledge of prenatal care, cash incentive scheme, utilization of received cash incentives, and respondents’ exposure to various SBCC (Social and Behavior Change Communication) inputs given under the program in the last three months.

The survey was part of periodic program monitoring and data/report of the survey were not published anywhere in public domain.

The selection of food items for diet diversity was contextualised based on the findings of a qualitative study conducted to understand food consumption patterns and the availability of food groups among the target population. To capture the consumption of various food items in the last 24 h (open recall), enumerators asked a series of listed food items in the tool to help the respondent recall all foods and beverages consumed in the last 24 h and probed for ingredients in mixed dishes. Each food or beverage that the respondent mentioned was punched on the predefined list by the enumerator.

### Study population & sampling

The target population for the cross-sectional survey was all pregnant women who had registered for nutrition counselling in the last six months from the reference date of the survey. The sample size was calculated using a population proportion determination formula based on the assumption of a 95% significance level, 4% margin of error, and 1.5 design effect, assuming prevalence for the unknown population 50% and 81% response rate. The calculated sample size was 6800 pregnant women in all five districts. 2000 Anganwadi Centers (AWCs) were selected as primary sampling units (PSUs) to achieve the sample size in each district. Simple random sampling was done while selecting the PSUs, assuming that at any point in one AWC, 5–6 pregnant women would be available. The number of AWCs per block was obtained by dividing the number of AWCs needed by the number of blocks in the districts.

### Variable description

#### Outcome variables

##### Minimum dietary diversity (MDD)

The MDD was calculated as a proxy indicator to understand the nutritional adequacy of pregnant women’s diet during pregnancy. This study defines MDD as consumption of food from at least five food groups out of 10 in the last 24 h. Based on recommendations of FAO, collected data on food consumption grouped broadly under 10 food groups: (a) Grains, white roots and tubers, plantains, ( b) Pulses (beans, peas and lentils), (c)nuts and seeds, (d) Dairy Products, (e) Meat, poultry and fish, (f) egg (g) Dark green leafy vegetables, (h) vitamin A- rich fruits and vegetables, (i) other vegetables, (j) other fruits (FAO, 2016). Consumption of oils and sweet dishes are excluded from the construction of MDD. A diet diversity score was calculated using the information on all these 10-food groups ranging from 0 to 10-point scale (“0” signifies non-consumption of any food items from food groups, and “10” signifies consumption of food from maximum food groups). Respondent scoring less than 5 was categorized as “not having minimum dietary diversity”, and more than 5 was categorized as “having minimum dietary diversity”. Hence, MDD was identified if a woman has consumed food from five or more food groups in the last 24 h.

#### Explanatory variables

##### Household Wealth Status

The household wealth status was assessed using information on ownership of livestock (goat, camel, cow/buffaloes, sheep, chicken or ducks), ownership of material assets (access to electricity, mobile phone, computer, refrigerator, pressure cooker, bicycle, car etc.), source of drinking water, type of toilet facilities, type of cooking fuel, type of material used in house’s floor, walls and roof, number of sleeping rooms, number of household members and ownership of bank account. Scores were assigned to each household based on the mentioned characteristics. The household’s score was calculated using principal component analysis (PCA) (Gausman, 2018). The score was divided into five equal groups: poorest, poorer, middle, richer, and richest, with 20% of the population in each group.

##### SBCC exposure

Exposure to various SBCC inputs was captured retrospectively, taking three-months reference period. In this study, SBCC exposure measures the intensity of program inputs. Household Visits by Poshan champion, Anganwadi Worker (AWW), Axillary Nurse Midwives (ANM) and counselling during MCHN day under the program were considered for calculating respondent’s exposure to SBCC. Respondents were asked about their exposure to these four touch points as well as the frequency of these touch points in the last three months. Based on the frequency of exposure to four touch points, each respondent was assigned a score ranging from 0 to 10 points scale, where “0” signifies no exposure at all and “10” signifies maximum exposure. Further categorization was done using this score as “0–2 low exposure”, “3–5 medium exposure” and “>5 high exposure”. Table 1 denotes the SBCC exposure touchpoints.

##### Received cash incentives

In targeted districts, two conditional cash transfer schemes are operational, referring to women’s first and second parity. Central government-funded Pradhan Mantri Matru Vandna Yojna (PMMVY) provides a conditional cash transfer of INR 6000 in case of first parity birth, and state-funded Indira Gandhi Matritva Poshan Yojna (IGMPY) provides a conditional cash transfer of INR 8000 for second parity pregnant women. The conditionalities of both schemes rely on early registration for ANC, ANC compliance and institutional Delivery. During the survey, respondents were asked “if they have received cash incentives under any of these schemes”. During analysis, the affirmative response was categorized as “1 beneficiary received cash incentives under any of schemes” “0 beneficiaries who did not receive cash incentives under any schemes”.

##### Belief in food-related myths

Considering the locally prevailing belief, information was captured on myths and taboos in the survey. During the survey, women were asked about their belief in myths related to restrictions on consuming certain food items, bananas, milk and Jaggery during pregnancy. Respondents who believed in any of these myths were categorized as “believing in food-related myths”, and respondents who answered “No” to all three myths-related questions were categorized as “do not believe in any food-related myth”.

##### Social characteristics

Caste was coded as General/Other backward caste (OBC), Schedule Caste (SC), and Schedule Tribe (ST). The study population has a higher proportion of SC and ST population. SC and ST caste are socially and economically disadvantaged groups and have faced historical discrimination in India. Religion was grouped as Hindu, Muslim and Other. The education level of women was categorized into four groups: “illiterate”, “Primary Education”, “Up to High School”, “Higher Secondary and Above”.

##### Other explanatory variable

The age of beneficiaries was re-categoriesed as “0 = < 25 years” and “1>=25 years”. During the survey, women were asked about their birth parity. Under the program, only first and second-parity beneficiaries are eligible for intervention. Hence, the analytical sample only has women of first and second parity. During the survey, women were asked about their knowledge of ideal ANC visits for a pregnant woman. For analysis, women’s response has been re-coded as “0 < 4 ANC visits” and “1 = > 4 ANC visits”.

### Statistical analysis

Descriptive statistics, including frequency distribution and cross-tabulations of each predictor and outcome variable, were used to describe variables for the study. Categorical variables were presented in percentages and frequency, whereas continuous variables were presented in mean and standard deviation. Pearson’s X2 test verified the association of the outcome variable with the predictors. Binary logistic regression was used to estimate odds ratios and 95% Confidence Intervals (CIs). Results were presented as crude odds ratio (cOR) and adjusted odds ratio (aOR) to assess the strength and presence of association, with a threshold of *p* < .05 used for determination of statistical significance.

Multivariate decomposition analysis was used to quantify the contribution of selected predictors in explaining the rich-poor gap in the prevalence of maternal dietary diversity. Multivariate decomposition technique uses the output from regression models to partition the components of a group difference in a statistic, such as a mean or proportion, into a component attributable to compositional differences between groups, i.e., differences in characteristics or endowments, and a component attributable to differences in the effects of characteristics, i.e., differences in the returns, coefficients or behavioural responses. The mean difference in Y between groups A and B can be decomposed as,

$$\begin{gathered}{Y_A} - {Y_B} = \overline {F\left( {{X_A}{\beta _A}} \right)} - \overline {F\left( {{X_B}{\beta _B}} \right)} = \hfill \\\,\,\,\,\,\,\underbrace {\left\{ {\overline {F\left( {{X_A}{\beta _A}} \right)} - \overline {F\left( {{X_B}{\beta _A}} \right)} } \right\}}_E + \underbrace {\left\{ {\overline {F\left( {{X_B}{\beta _A}} \right)} - \overline {F\left( {{X_B}{\beta _B}} \right)} } \right\}}_C \hfill \\ \end{gathered}$$

where Y denotes the *N* × 1 dependent variable vector, X is an N × K matrix of independent variables, and β is a K × 1 vector of the coefficient. The component labelled E refers to the part of the differential attributable to differences in endowments or characteristics, usually called the explained component or characteristics effects. C refers to the part of the differential attributable to differences in coefficients or effects usually known as the unexplained component or coefficient effects, where A is the pregnant woman from the richest household (comparison group), and B is the pregnant woman from the poorest household (reference group). Therefore, E reflects a counterfactual comparison of the difference in outcomes from the women from the richest household perspective, and C reflects a counterfactual comparison of outcomes from women from the poorest household perspective. STATA 18.0 software was used for data analysis.

## Results

### Socio-demographic characteristics

Table 2 depicts the descriptive characteristics of the participants. A total of 6848 participants were surveyed during May-June 2023. The mean (+/- Standard deviation (SD)) age of the respondents was 23.0 (+/-3.8) years and more than 70% of the women were in the age group of < 25 years. Most women (97.1%) belong to the Hindu religion, and around two-third of them belong to schedule caste or scheduled tribe community. 14.8% of pregnant women attended primary school based on their educational status. Less than three-fourth (72.7%) of the women had correct knowledge about the number of Antenatal checkups (ANC) visits, and around 39% had pregnancy food-related myths.

**Table 2:** Socio-demographic characteristics of the pregnant women

Background Characteristics	Number (*n*)	Percent (%) (95% CI)
**Maternal Characteristics**		
**Age (years), Mean ± Standard Deviation (SD)**	23(3.8)	
Age		
< 25	4874	71.2(70.1–72.2)
>=25	1974	28.8(27.8–29.9)
**Caste**		
General /OBC	2287	33.4(32.3–34.5)
SC	1509	22.0(21.1–23)
ST	3052	44.6(43.4–45.7)
**Education**		
No formal education	823	12.0(11.3–12.8)
Primary	1011	14.8(13.9–15.6)
Up to highschool	3172	46.3(45.1–47.5)
Senior secondary & above	1842	26.9(25.9–28.0)
**Household Wealth Status**		
Poorest	1370	20.0(19.1–21.0)
Poorer	1370	20.0(19.1–21.0)
Middle	1369	19.9(19.1–21.0)
Rich	1370	20.0(19.1–21.0)
Richest	1369	19.9(19.1–21.0)
**Knowledge about ANC visits**		
< 4 visits	1868	27.3(26.2–28.3)
4 or more visits	4980	72.7(71.7–73.8)
**Parity of Women**		
First	4940	72.1(71.1–73.2)
Second	1908	27.9(26.8–28.9)
**Belief in food related myths**		
No	4200	61.3(60.2–62.5)
Yes	2648	38.7(37.5–39.8)

### Program exposure at individual and community level

Table 3 presents the pregnant women’s exposure to various SBCC program interventions. Around 43% of the pregnant women reported receiving cash incentives, and two-third (67%) reported receiving counselling by a Poshan Champion during home visits in the last three months (Table 2). Furthermore, 75.4% reported being visited by ANM and 79.5% by AWW. Over half (56.1%) attended a community-level Maternal Child Health and Nutrition Day in the last three months. Regarding social and behaviour change (SBCC) intervention exposure, less than one-fourth (23.6%) of the pregnant women had a low exposure, whereas around 33.7% reported a high SBCC exposure index.

**Table 3:** Individual and community level program exposure of pregnant women

Exposure to Program Interventions	Number (*n*)	Percent (%) (95% CI)
**Received cash incentive**		
No	3901	57.9(56.2–58.6)
Yes	2947	43.0(41.4–43.8)
**Received counselling by ANM in last three months**		
No	1684	24.6(23.6–25.6)
Yes	5164	75.4(74.4–76.4)
**Received counselling by AWW in last three months**		
No	1407	20.6(19.0-21.5)
Yes	5441	79.5 (78.4–80.4)
**PC visited for counselling in last three months**		
No	2264	33.0(31.8–34.2)
Yes	4584	66.9(65.8–68.1)
**Attended MCHN Day in last three months**		
**No**	3034	43.7(42.7–45.1)
Yes	3814	56.1(54.9–57.3)
SBCC Exposure*		
Low	1614	23.6(23.1–25.6)
Medium	2928	42.8(42.0-44.1)
High	2306	33.7(33.1–35.4)

*SBCC Exposure is defined as the frequency of interaction at each of the SBCC touchpoints namely (ANM, AWW, PCs, MCHN day)

### Dietary diversity status

Table 4 depicts consumption of individual food groups and minimum dietary diversity among pregnant women. More than half (55.2%) of the pregnant women reported consumption of a diverse diet (Table 4). The mean dietary diversity score was 4.8, with SD +/- 1.5. Regarding the consumed food groups by pregnant women in the previous 24 h, more than (95%) of the pregnant women consumed grains, white root, tuber, and pulses. In addition, around (83%) reported consumption of pulses beans and lentils. Among the animal source foods, only 2.6% consumed flesh food and eggs, whereas around two-thirds reported consumption of milk and milk products. Less than half of the pregnant women consumed vitamin A rich foods and vegetables, and 38.3% reported consumption of green leafy vegetables.

**Table 4:** Dietary Diversity and individual food group consumption by pregnant women

Food Groups	Number (*n*)	Percent (%) (95%CI)
Grains, white roots, tubers, and plantains	6552	95.8(95.2–96.2)
Pulses (beans, peas, and lentils)	5734	83.6(82.7–84.5)
Nuts and Seeds	291	4.5(4.0-5.1)
Dairy	4541	66.4(65.2–67.6)
Meat, poultry, and fish	22	0.3(0.2–0.5)
Eggs	158	2.3(2.0-2.7)
Dark green leafy vegetables	2666	38.3(38.1–40.5)
Other Vitamin A-rich fruits and vegetables	3238	47.3(46.1–48.5)
Other vegetables	6343	92.6(91.9–93.2)
Other fruits	3331	48.9(47.7–50.1)
**Dietary diversity status**		
Diverse (%)	3768	55.2(54.0-56.4)
Not diverse (%)	3080	44.8(43.6–46.0)
Mean +/- SD	**4.8 (1.5)**	

#### Factors associated with dietary diversity during pregnancy

Table 5 presents the bivariate and multivariate regression analysis. In bivariate logistic regression analysis, the program exposure variables, counselling received from the Poshan champion, FLWs and participating in the MCHN day in the last three months were positively associated (P-value less than 0.05) with increasing odds of maternal minimum dietary diversity. Across the socio-demographic variables, respondent age group (more than 25 years), education, and richest economic status were positively associated, whereas belonging to a scheduled caste or tribe and knowledge of ANC visits were negatively associated with maternal dietary diversity. In multivariable logistic regression analysis, the adjusted odds of consuming a diverse diet by pregnant women in model 1 was 1.1 times (aOR = 1.1, 95% CI: 1.0-1.3) and 2.7 times (aOR = 2.7, 95% CI: 2.4–3.1) higher among those who received a medium and high-level SBCC program exposure respectively compared to those who received a low level of exposure (Table 5). Pregnant women from wealthier households and those with a higher secondary and above level of education were 1.7 times more likely to consume a diverse diet than the women from low-wealth households and with no formal education (aOR = 1.4, 95% CI: 1.2–1.7) and higher education (aOR = 1.1, 95% CI: 0.9-1.3) respectively. Women belonging to scheduled caste or scheduled tribe households were 0.7 times less likely (aOR = 0.7, 95% CI: 0.6–0.8) to consume a diverse diet than those belonging to general caste households. Similarly, women who reported having the correct knowledge on the ANC visits were less likely to consume a diverse diet (aOR = 0.7, CI: 0.6–0.9). The odds of pregnant women consuming a diverse diet among those receiving cash incentives was 0.8 times less likely (aOR = 0.8, CI:0.7–0.9) than their counterparts. In model 2, the adjusted odds of pregnant women consuming a diverse diet were 1.3 times (aOR = 1.3, CI: 1.1–1.4), 1.9 times (aOR = 1.9, CI: 1.7–2.1), and 1.0 times (aOR = 1.0, CI: 0.9-1.2) more likely when received counselling by frontline workers, community motivators and participation in the MCHN day, respectively.

**Table 5:** Bivariate and multivariate binary logistic regression of maternal and socio demographic factors associated with dietary diversity among pregnant women

Exposure to Program Inputs & Background Characteristics	Dietary Diversity		Bivariate Analysis	Multivariate Analysis (Model1)	Multivariate Analysis (Model2)	
	Not Diverse (%)	Diverse (%)	cOR (*p*-value) (95% CI)	aOR (*p*-value) (95% CI)	aOR (*p*-value) (95% CI)	
**Visited by AWW in last three months**						
No	49.0	51.0	Ref	-	Ref	
Yes	43.9	56.1	1.2**(1.0-1.4)	-	0.9(0.8–1.1)	
**Visted by ANM in last three months**						
No	53.1	46.9	Ref	-	Ref	
Yes	42.3	57.7	1.5***(1.4–1.7)	-	1.3***(1.1–1.4)	
**Visited by PC for counselling in last three months**						
No	56.3	43.7	Ref	-	Ref	
Yes	39.4	60.6	2.0***(1.8–2.2)	-	1.9***(1.7–2.1)	
**Attended MCHN day in last three months**				-		
No	48.1	51.9	Ref	-	Ref	
Yes	42.5	57.5	1.2***(1.1–1.4)	-	1.0**(0.9–1.2)	
**Received cash incentive**						
No	41.9	58.1	Ref	Ref	-	
Yes	48.8	51.2	0.7***(0.7–0.8)	0.8***(0.7–0.9)	-	
**SBCC Exposure**						
Low	54.7	45.3	Ref	Ref	-	
Medium	51.3	48.7	1.1**(1.0-1.3)	1.1**(1.0-1.3)	-	
High	30.2	69.8	2.8***(2.4–3.2)	2.7***(2.4–3.1)	-	
**Belief in food related myths**					-	
No	43.8	56.2	Ref	Ref	-	
Yes	46.5	53.6	0.8**(0.8–0.9)	0.9(0.8-1.0)	-	
**Knowledge of ANC visits**					-	
< 4 visits	39.9	60.1	Ref	Ref	-	
4 or more visits	46.7	53.3	0.7***(0.6–0.8)	0.7***(0.6–0.9)	-	
**Parity of women**					-	
First	43.8	56.3	Ref	Ref	-	
Second	47.6	52.4	0.9* **(0.8–0.9)	0.8**(0.7-0.8)	-	
**Age-groups (in years)**			-	-	-	
< 25	46.3	53.7	Ref	Ref	-	
>=25	41.7	58.3	1.2***(1.1–1.3)	1.0(0.9–1.2)	-	
**Caste**				-	-	
Other	36.6	63.4	Ref	Ref	-	
SC	44.7	55.3	0.7***(0.6–0.8)	0.8**(0.7–0.9)	-	
ST	51.3	48.7	0.5***(0.5–0.6)	0.7***(0.6–0.8)	-	
**Education**					-	
No formal Education	52.1	47.9	Ref	Ref	-	
Primary	41.4	58.6	1.5***(1.2–1.8)	1.4***(1.2–1.7)	-	
Up to high school	47.5	52.5	1.2**(1.0-1.4)	1.0(0.8–1.2)	-	
Higher Secondary and above	39.1	60.9	1.6***(1.4-2.0)	1.1(0.9–1.3)	-	
**Household Wealth Status**					-	
Poorest	48.1	51.9	Ref	Ref	-	
Poorer	51.5	48.5	0.9(0.8–1.1)	0.8**(0.7-1)	-	
Middle	50.0	50.0	0.9(0.8–1.1)	0.8(0.8–1.1)	-	
Rich	45.8	54.2	1.1**(1.0-1.4)	1.1(0.9–1.4)	-	
Richest	29.4	70.6	2.4***(2.0-2.8)	1.7***(1.5–2.3)	-	

cOR Unadjusted odds ratio; aOR Adjusted odds ratio

*** *p* < .01, ** *p* < .05, * *p* < .1

*Model 1 effect of SBCC exposure on MDD after controlling for i.e. age of women, education level of women, household wealth status, caste, parity of women and knowledge of ANC

*Model 2 effect of individual SBCC touchpoint on MDD after controlling for i.e. age of women, education level of women, household wealth status, caste, parity of women and knowledge of ANC

### Socio-economic inequality and decomposition of factors

There was around a 19-percentage point difference in diverse diet consumption among the pregnant women belonging to the lowest and highest wealth quantile households. The results of the multivariable regression-based decomposition analysis for the socio-economic inequality of maternal dietary diversity are shown in Table 6. The decomposition analysis shows how the endowment and coefficient effects contribute to the gap in the maternal dietary diversity prevalence among women between the lowest and highest wealth quantile households. A negative contribution indicates that the determinant narrowed the gap between the lowest and highest wealth quantiles households and vice-versa. Differences due to coefficient accounted for 57.95% of the observed socio-economic differential in the prevalence of maternal dietary diversity, and the difference due to characteristics accounted for 42.05%. The difference explained by characteristics is further explained by contribution from various covariates. For instance, if the mothers from the poorest households were as educated as those from the richest households, the prevalence of a non-diversified diet among pregnant women would reduce by 18.48%. Similarly, narrowing the caste-based differentials would reduce the gap by 22.41%. The difference in exposure to SBCC interventions among pregnant women from the poorest and richest households contributed to a gap of 7.80% in the prevalence of dietary diversity.

**Table 6:** Decomposition of difference in prevalence of diversified diet among pregnant women between poor and rich households

Exposure to Program Inputs & Background Characteristics	Difference due to characteristics (Explained)			Difference due to coefficients (Unexplained)		
	Coefficient	%	*p*-value	Coefficient	%	*p*-value
Women’s age	0.006	3.1	0.409	0.008	4.1	0.269
Caste	0.042	22.4	0.026	0.056	29.9	0.107
Educational status	0.035	18.5	0.089	0.051	27.4	0.307
Knowledge of ANC visit	-0.006	-3.3	0.004	0.004	2.2	0.884
Belief in food related myths	-0.0001	-0.1	0.771	-0.05	-26.7	0.002
SBCC Exposure	0.014	7.6	< 0.001	0.106	56.8	< 0.001
Received cash incentives	-0.011	-6.2	0.007	-0.008	-4.0	0.409
Constant				-0.059	-31.8	0.505
**Total**	0.078	42.1	0.006	0.108	57.9	0.001

## Discussion

The current study contributes to a limited but growing literature on maternal dietary diversity, associated socio-economic factors, impact of social behaviour change interventions and factors contributing to socio-economic inequality. The findings indicate that more than half (55.2%) of the pregnant women in the selected five districts of Rajasthan had met a minimum dietary diversity. Further, the mean dietary diversity score in the study was reported as 4.8 +/- 1.5. To our knowledge, this is the first study in the selected study sites. A study conducted in Haryana by [22] reported more than three-fourth of the lactating mothers consuming a minimum diversified diet. Compared to this study, the high prevalence of dietary diversity can be attributed to the fact that the state of Haryana is predominantly an agricultural state, resulting in relatively better dietary diversity [23].

Further, the difference in the prevalence of minimum dietary diversity can be attributed to the seasonal differential, i.e., period when data was collected, socio-cultural factors and overall profile of the study population. According to the Rajasthan state-specific Sustainable Development Goal (SDG) index, all five selected districts for this study were among the bottom-ranked 10 districts of Rajasthan [24]. It can also be associated with a higher prevalence of tribal population in the region, leading to protracted socio-economic inequality in the region.

In this study, about (96%) of pregnant women consumed grain, white roots, tubers, and plantation food groups. The findings corroborate the study done in Pune, Maharashtra [25] reporting high starchy staple food group consumption. Less than (3%) of pregnant women reported consumption of food groups such as meat, poultry, fish, and eggs. This is in alignment with the study on dietary intake among pregnant women in India [26] and the fact that most of the population in Rajasthan prefers vegetarian diets [27] Two-third of the respondents reported consumption of dairy products. High consumption of dairy products may be attributed to the increased purchasing power of pregnant women from the cash incentive received during pregnancy. Supplementary Table 1 shows the distribution of food groups across those with a diverse and non-diverse dietary diet among the poorest and richest wealth quintile groups. The findings state that pregnant women from both the wealth quintiles consume dairy products, which are cost intensive. The study conducted among pregnant women in Nepal reported higher proportions of cash incentives for purchasing and consuming milk [28]. These findings align with the current study, indicating that cash availability improves the purchasing power for expensive micronutrient rich food with lesser calorie value across socio-economic groups [29]. Vitamin A rich fruits and vegetables and dark green vegetables were consumed by (39%) and (47%) of pregnant women, viz. The relatively high consumption of dark green vegetables, vitamin A-rich fruits and vegetables, and starchy staple food groups is a distinct pattern and does not concur with previous studies. This may be attributed to increased knowledge and awareness of maternal nutrition and diet diversification due to specific messaging from the frontline workers and Poshan champions who delivered targeted, tailored messages highlighting the importance of same.

Factors associated with consumption of specific food groups to achieve a minimum dietary diversity are multi-dimensional. The study findings indicate that pregnant women from wealthier households were more likely to consume a diverse diet than women from poorer households. The findings were corroborated by prior studies done in the domain [17, 30, 31]. These findings support Bennett’s law, which states that with greater resources, there is relocation among food groups constituting diverse diet and consumption of improved and nutritious diets [32]. This is also supported by our study that shows that despite a lesser share in the consumption of pulses, dark and green leafy vegetables, Vit A rich fruits and other fruits among dietary diversified women in the richest wealth quintile, they are more likely to consume diversified food items than women in poorest wealth quintile.

Besides the association of dietary diversity with economic factors, dietary diversity was positively associated with educational status, where pregnant women with higher education had a more diverse diet. This finding aligns with previous studies conducted in Uttar Pradesh [17] and a multi-state study done in Bihar, Chhattisgarh, and Odisha [33]. One of the possible explanations could be that women with higher education levels have better exposure and, therefore, are more likely to receive appropriate information related to diet diversity and comprehend the importance of maternal nutrition [34, 35]. Additionally, education can be seen as a proximate determinant of women’s empowerment, allowing women to exercise their rights related to household and individual level decisions, particularly on the financial allocation for the purchase of nutrient-dense food [36, 37]. Further, socio-cultural factors such as the caste of the pregnant women was found to be associated with their diet diversity. Women belonging to the Scheduled caste and Scheduled tribe have lower dietary variety than those belonging to general and other backward classes. These findings are consistent with the past study [38] explaining the role of economic and social status on women’s nutritional status in India. Women from socially disadvantaged castes face multifaceted challenges such as lack of knowledge, social taboos, limited individual agency and decision-making power [39–43]. This leads to poor diet diversification and micronutrient deficiency during pregnancy, which has acute and chronic repercussions on women and their offspring’s health [44].

Food-related myths and perceived restrictions during pregnancy were not significantly associated with maternal dietary diversity. This contrasts with previous studies’ findings [45, 46], where, food-related taboos were associated with dietary restrictions. Several food items were believed to be heat-producing, deforming the fetus’s growth, and therefore, should be restricted during pregnancy. The difference in findings of our study can be related to targeted messaging on the importance of diet diversification and busting the myths and taboos related to several food items within the communication package of the program delivered by the Poshan Champions and frontline workers at every stage of pregnancy. In addition, this can also be attributed to the fact that women find alternative food items to replace the restricted food items. Future research to understand the role of family members, particularly understanding Mother-in-law’s perspective on the food-related myths during pregnancy, will be informative.

Various strategies and platforms under social and behavioural change communication (SBCC) were adopted under the Rajpusht project to improve knowledge and awareness of maternal nutrition. Regression analysis in Table 5 shows that maternal dietary diversity is more likely to be higher among pregnant women who received interpersonal counselling and attended community-based events through PCs, ANMs, AWWs and MCHN days. While PCs and AWWs provide regular counselling, support, and monitoring of the women during pregnancies, they also promote awareness among women and their families on diets, supplementary nutrition, vaccinations, and breaking common myths in the community. One key message communicated by ANMs and then supported by PCs during IPC concerns the importance of weight gain during pregnancy. ANMs are specifically capacitated under the programme to provide intensive counselling to pregnant women on vital messages and awareness of localised diverse diets via specialised job aids developed as part of the SBCC strategy. It is important to discuss that the RajPusht intervention has been programmed uniquely by incorporating multiple SBCC touch points at regular intervals, ensuring a comprehensive yet high-dose response to the targeted women, their families, and communities. Multiple modes of social and behavioural change interventions aimed at improving maternal and child health have been recognised to generate positive outcomes in multiple settings [47]. Improvement in outcome indicators like maternal dietary diversity can be envisaged by efficient program coverage to identify pregnant women and expose them to behavioural change interventions [48]. Our study substantiates that achieving equity in the health indicators mostly depends on the dose-response and reach of the population. Compliance towards program intervention is better for those with higher socio-economic status due to better access to resources and services. It subsequently diffuses to the lower socio-economic groups; however, diffusion’s intensity can be time-dependent [49].

SBCC interventions and cash incentives can have a long-term impact beyond program due duration than only cash interventions by modifying immediate and underlying factors of malnutrition [50, 51]. Studies have shown that cash incentives play a role in purchasing food items that are perceived as costly or not purchased by households regularly [52]. Our study also aligns with this argument and provides substantial evidence that cash incentives promote a higher purchase power for protein-rich items among our targeted population.

Socio-economic inequality in the consumption of diverse dietary products can be well explained through the multivariate decomposition analysis done in the study. The study findings revealed that 42% of the gap in the prevalence of diversified diets among pregnant women was attributed mainly to the distribution of determinants between rich and poor households. Our model provides evidence for discernible divergence in dietary diversity among the poorest and richest wealth quintiles through caste and educational attainment of the women. Better accessibility of knowledge and addressing the interlinked detrimental aspects of lower socio groups are vital for improved nutritional practices among women. Therefore, the role of social and behavioural change interventions aiming to improve knowledge, awareness and building of an enabling environment in the community are critical to have positive, sustained behavioural change.

The present study findings should be seen under certain limitations. Firstly, dietary diversity was determined based on answers from participants’ recalls, which might have some recall bias. Secondly, the cross-sectional nature of the data limits causal inference between the outcome and predictor variables. Thirdly, as dietary diversity depends on readily and locally available food items, seasonality might have played some role, which may limit the generalizability of findings to other seasons. Lastly, the study is limited to measuring the combinations of food groups consumed by the women and the most prevalent combinations consumed by the women in the study area. Further study can shed light on building such combinations to measure the best dietary combinations for the birth outcomes of pregnant women.

## Conclusion

The study finding reveals that there is association between socio-economic characteristics of pregnant women and consumption of minimum dietary diversity (MDD). Improving women’s education level, exposure to SBCC interventions (nutrition specific) contribute to higher dietary diversity during pregnancy. Despite a predominant vegetarian diet consuming population, better maternal dietary diversity was observed among those exposed to higher dose of SBCC intervention package. Educational status and caste of the respondent were significantly associated with minimum dietary diversity and contributed to the socio-economic inequality highlighting the importance of tailored and sustained SBCC interventions.

### Electronic supplementary material

Below is the link to the electronic supplementary material.

Supplementary Material 1

Supplementary Material 2

## Data Availability

Reasonable request for data used in this article may be made to the corresponding author.

## Abbreviations

SBCC: Social and Behavioural Change Communication
MDD: Minimum Dietary Diversity
NM: Neonatal Mortality
LBW: Low Birth Weight
BMI: Body Mass Index
CIFF: Children’s Investment Fund Foundation
PC: Poshan Champion
MCHND: Maternal, Child Health and Nutrition Day
ANM: Auxiliary Nurse and Midwife
AWW: Anganwadi Workers
IGMPY: Indira Gandhi Matritva Poshan Yojna
PMMVY: Pradhan Mantri Matru Vandna Yojna

## References

[1] Tette EMA, Intiful FD, Asare AA, Enos JY (2022). Pregnancy as a Fundamental Determinant of Child Health: a review. Curr Nutr Rep.

[2] Dandona R, Kumar GA, Henry NJ, Joshua V, Ramji S, Gupta SS (2020). Subnational mapping of under-5 and neonatal mortality trends in India: the global burden of Disease Study 2000–17. Lancet.

[3] Coffey D (2015). Prepregnancy body mass and weight gain during pregnancy in India and sub-saharan Africa. Proc Natl Acad Sci U S A.

[4] Abu-Saad K, Fraser D. Maternal nutrition and birth outcomes. Epidemiologic Reviews. Volume 32. Oxford University Press; 2010. pp. 5–25. 10.1093/epirev/mxq001 20237078

[5] Swaminathan S, Hemalatha R, Pandey A, Kassebaum NJ, Laxmaiah A, Longvah T (2019). The burden of child and maternal malnutrition and trends in its indicators in the States of India: the global burden of Disease Study 1990–2017. Lancet Child Adolesc Health.

[6] Blencowe H, Krasevec J, de Onis M, Black RE, An X, Stevens GA (2019). National, regional, and worldwide estimates of low birthweight in 2015, with trends from 2000: a systematic analysis. Lancet Glob Health.

[7] WHO. Ambition and action in nutrition 2016–2025. 2017.

[8] FAO, FHI 360. Minimum Dietary Diversity for Women- A Guide to Measurement [Internet]. Rome. 2016. Available from: www.fao.org/publications.

[9] Gupta S, Sunder N, Pingali PL (2020). Are women in rural India really consuming a less diverse Diet?. Food Nutr Bull.

[10] Sims A, van der Pligt P, John P, Kaushal J, Kaur G, McKay FH. Food insecurity and dietary intake among rural Indian women: an exploratory study. Int J Environ Res Public Health. 2021;18(9). 10.3390/ijerph18094851 PMC8124183 34062823

[11] Demilew YM, Alene GD, Belachew T. Dietary practices and associated factors among pregnant women in West Gojjam Zone, Northwest Ethiopia. BMC Pregnancy Childbirth. 2020;20(1). 10.1186/s12884-019-2702-z PMC6945405 31906981

[12] Diemert A, Lezius S, Pagenkemper M, Hansen G, Drozdowska A, Hecher K et al. Maternal nutrition, inadequate gestational weight gain and birth weight: results from a prospective birth cohort. BMC Pregnancy Childbirth. 2016;16(1). 10.1186/s12884-016-1012-y PMC4986204 27528213

[13] Lee SE, Talegawkar SA, Merialdi M, Caulfield LE (2013). Dietary intakes of women during pregnancy in low- and middle-income countries. Public Health Nutr.

[14] Stevens GA, Finucane MM, De-Regil LM, Paciorek CJ, Flaxman SR, Branca F et al. Global, regional, and national trends in haemoglobin concentration and prevalence of total and severe anaemia in children and pregnant and non-pregnant women for 1995–2011: a systematic analysis of population-representative data. Lancet Glob Health. 2013;1(1). 10.1016/S2214-109X(13)70001-9 PMC4547326 25103581

[15] Sharma S, Akhtar F, Singh RK, Mehra S. Dietary Intake across Reproductive Life Stages of Women in India: A Cross-Sectional Survey from 4 Districts of India. Vol. 2020, Journal of Nutrition and Metabolism. Hindawi Limited; 2020. 10.1155/2020/9549214 PMC7341409 32685210

[16] Kheirouri S, Alizadeh M. Maternal dietary diversity during pregnancy and risk of low birth weight in newborns: a systematic review. Public Health Nutrition. Volume 24. Cambridge University Press; 2021. pp. 4671–81. 10.1017/S1368980021000276 PMC10195329 33472725

[17] Rammohan A, Goli S, Singh D, Ganguly D, Singh U (2019). Maternal dietary diversity and odds of low birth weight: empirical findings from India. Women Health.

[18] Barker M, Dombrowski SU, Colbourn T, Fall CHD, Kriznik NM, Lawrence WT, et al. Intervention strategies to improve nutrition and health behaviours before conception. The Lancet. Volume 391. Lancet Publishing Group; 2018. pp. 1853–64. 10.1016/S0140-6736(18)30313-1 PMC6075694 29673875

[19] Habtu M, Agena AG, Umugwaneza M, Mochama M, Munyanshongore C. Effect of integrated nutrition-sensitive and nutrition-specific intervention package on maternal malnutrition among pregnant women in Rwanda. Matern Child Nutr. 2022;18(3). 10.1111/mcn.13367 PMC9218321 35538044

[20] Houweling TAJ, Kunst AE, Huisman M, Mackenbach JP. Using relative and absolute measures for monitoring health inequalities: experiences from cross-national analyses on maternal and child health. Int J Equity Health. 2007;6. 10.1186/1475-9276-6-15 PMC2173893 17967166

[21] Bhatia M, Dwivedi LK, Banerjee K, Bansal A, Ranjan M, Dixit P. Pro-poor policies and improvements in maternal health outcomes in India. BMC Pregnancy Childbirth. 2021;21(1). 10.1186/s12884-021-03839-w PMC8135986 34011316

[22] Shumayla S, Irfan EM, Kathuria N, Rathi SK, Srivastava S, Mehra S. Minimum dietary diversity and associated factors among lactating mothers in Haryana, India: a community based cross-sectional study. BMC Pediatr. 2022;22(1). 10.1186/s12887-022-03588-5 PMC9440519 36057585

[23] Singh S, Jones AD, Jain M. Regional differences in agricultural and socioeconomic factors associated with farmer household dietary diversity in India. PLoS ONE. 2020;15(4). 10.1371/journal.pone.0231107 PMC7161949 32298281

[24] Center for SDGs Implementation. Government of Rajasthan Rajasthan Sustainable Development Goals. Jaipur, Rajasthan; 2023.

[25] Gokhale D, Rao S. Socio-economic and socio-demographic determinants of diet diversity among rural pregnant women from Pune, India. BMC Nutr. 2022;8(1). 10.1186/s40795-022-00547-2 PMC9254638 35787284

[26] Sharma S, Akhtar F, Kumar Singh R, Mehra S. Dietary patterns and determinants of pregnant and Lactating women from marginalized communities in India: A Community-based cross-sectional study. Front Nutr. 2020;7. 10.3389/fnut.2020.595170 PMC7691489 33282903

[27] International Institute for Population Sciences (IIPS), ICF. National Family Health Survey (NFHS-5), 2019-21 [Internet]. Mumbai. 2021. http://www.rchiips.org/nfhs.

[28] Gram L (2018). Women’s empowerment in a complex public health intervention in rural Nepal.

[29] Harris-Fry HA, Paudel P, Harrisson T, Shrestha N, Jha S, Beard BJ (2018). Participatory women’s groups with cash transfers can increase dietary diversity and micronutrient adequacy during pregnancy, whereas women’s groups with food transfers can increase equity in intrahousehold energy allocation. J Nutr.

[30] Nguyen PH, Kachwaha S, Avula R, Young M, Tran LM, Ghosh S et al. Maternal nutrition practices in Uttar Pradesh, India: role of key influential demand and supply factors. Matern Child Nutr. 2019;15(4). 10.1111/mcn.12839 PMC6852235 31066195

[31] Mukhopadhyay S, Sarkar A (2009). Pregnancy-related food habits among women of rural Sikkim, India. Public Health Nutr.

[32] Cirera X, Masset E. Income distribution trends and future food demand. Vol. 365, Philosophical Transactions of the Royal Society B: Biological Sciences. Royal Society; 2010. pp. 2821–34. 10.1098/rstb.2010.0164 PMC2935126 20713387

[33] Unisa S, Saraswat A, Bhanot A, Jaleel A, Parhi RN, Bhattacharjee S et al. Predictors of the diets consumed by adolescent girls, pregnant women and mothers with children under age two years in rural eastern India. J Biosoc Sci. 2020. 10.1017/S0021932020000462 32782055

[34] Raghupathi V, Raghupathi W. The influence of education on health: an empirical assessment of OECD countries for the period 1995–2015. Archives Public Health. 2020;78(1). 10.1186/s13690-020-00402-5 PMC7133023 32280462

[35] Weitzman A (2017). The effects of women’s education on maternal health: evidence from Peru. Soc Sci Med.

[36] The Lancet Public Health. Education: a neglected social determinant of health. Vol. 5, the Lancet Public Health. Elsevier Ltd; 2020. p. e361. 10.1016/S2468-2667(20)30144-4 PMC7326385 32619534

[37] Chatterjee K, Sinha RK, Kundu AK, Shankar D, Gope R, Nair N et al. Social determinants of inequities in under-nutrition (weight-for-age) among under-5 children: a cross sectional study in Gumla district of Jharkhand, India. Int J Equity Health. 2016;15(1). 10.1186/s12939-016-0392-y PMC4938901 27391031

[38] Dahiya S, Viswanathan B (2015). Women’s malnutrition in India: the role of Economic and Social Status. Margin J Appl Econ Res.

[39] Ghosal J, Bal M, Ranjit M, Das A, Behera MR, Satpathy SK et al. To what extent classic socio-economic determinants explain trends of anaemia in tribal and non-tribal women of reproductive age in India? Findings from four National Family Heath surveys (1998–2021). BMC Public Health. 2023;23(1). 10.1186/s12889-023-15838-x PMC10172723 37170116

[40] Gonmei Z, Toteja GS. Micronutrient status of Indian population. Vol. 148, Indian Journal of Medical Research. Wolters Kluwer Medknow Publications; 2018. pp. 511–21. 10.4103/ijmr.IJMR_1768_18 PMC6366258 30666978

[41] Rao N, Caste (2014). Kinship, and Life Course: rethinking women’s work and Agency in Rural South India. Fem Econ.

[42] Sahoo M, Pradhan J (2021). Reproductive health care status of the displaced tribal women in India: an analysis using Nussbaum Central human capabilities. Health Care Women Int.

[43] Thorat S. Social Exclusion in the Indian context: Theoretical Basis of Inclusive Policies.

[44] Mahapatro SR, James KS, Mishra US. Intersection of class, caste, gender and unmet healthcare needs in India: implications for health policy. Health Policy Open. 2021;2. 10.1016/j.hpopen.2021.100040 PMC10297749 37383501

[45] Chakrabarti S, Chakrabarti A (2019). Food taboos in pregnancy and early lactation among women living in a rural area of West Bengal. J Family Med Prim Care.

[46] G Laksmi (2013). Food preferences and taboos during ante-natal period among the tribal women of north coastal Andhra Pradesh. J Community Nutr Health.

[47] Workicho A, Biadgilign S, Kershaw M, Gizaw R, Stickland J, Assefa W et al. Social and behaviour change communication to improve child feeding practices in Ethiopia. Matern Child Nutr. 2021;17(4). 10.1111/mcn.13231 PMC8476421 34132054

[48] Biswas T, Townsend N, Magalhaes R, Hasan MM, Al Mamun A. Geographical and socioeconomic inequalities in the double burden of malnutrition among women in Southeast Asia: A population-based study. The Lancet Regional Health - Southeast Asia [Internet]. 2022;1:100007. https://doi.org/10.1016/j. 10.1016/j.lansea.2022.04.003 PMC10305935 37383092

[49] Nickel S, Von Dem Knesebeck O. Do multiple community-based interventions on health promotion tackle health inequalities? International Journal for Equity in Health. Volume 19. BioMed Central Ltd; 2020. 10.1186/s12939-020-01271-8 PMC7488049 32912257

[50] Maffioli EM, Headey D, Lambrecht I, Oo TZ, Zaw NT (2023). A Prepandemic Nutrition-Sensitive Social Protection Program has sustained benefits for Food Security and Diet Diversity in Myanmar during a severe Economic Crisis. J Nutr.

[51] Ruel MT, Quisumbing AR, Balagamwala M. Nutrition-sensitive agriculture what have we learned and where do we go from here? [Internet]. Washington, DC; 2017 Oct. Electroniccopyavailableat: https://ssrn.com/abstract=3044587.

[52] Rao KD, Kachwaha S, Kaplan A, Bishai D. Not just money: what mothers value in conditional cash transfer programs in India. BMJ Glob Health. 2020;5(10). 10.1136/bmjgh-2020-003033 PMC7580051 33087391

