# Supplementary material for: Effect of social and behavioral change interventions on minimum dietary diversity among pregnant women and associated socio-economic inequality in Rajasthan, India
Source: BMC Nutr. 2024 Jun 6;10:82. doi: 10.1186/s40795-024-00887-1 (PMC11154971; doi:10.1186/s40795-024-00887-1)
Supplement: Supplementary file 1 — Supplementary Material 1 [file 40795_2024_887_MOESM1_ESM.docx]

**Supplementary Table**

**Table 1: Consumption of various food groups among Rich and Poor Women (annexure)**

|  | **Poorest** | | **Richest** | |
| --- | --- | --- | --- | --- |
| **Food Groups** | **Not Diverse Diet (%)** | **Diverse Diet (%)** | **Not Diverse Diet (%)** | **Diverse Diet (%)** |
| **Grains, white roots and tubers, and plantains** | 99.2 | 100.0 | 94.5 | 99.9 |
| **Pulses (beans, peas and lentils)** | 80.1 | 99.0 | 52.4 | 92.8 |
| **Nuts and Seeds** | 0.0 | 2.1 | 1.5 | 13.5 |
| **Dark green leafy vegetables** | 11.2 | 78.8 | 17.4 | 64.6 |
| **Other Vegetables** | 85.3 | 99.4 | 82.9 | 99.6 |
| **Other Vitamin A-rich fruits and vegetables** | 14.1 | 77.8 | 14.4 | 70.0 |
| **Other Fruits** | 10.3 | 81.3 | 24.1 | 77.7 |
| **Dairy** | 25.6 | 80.2 | 57.8 | 89.4 |
| **Meat, Poultry and fish** | 0.2 | 0.1 | 0.0 | 0.2 |
| **Eggs** | 0.5 | 5.6 | 0.3 | 3.0 |
