# Supplementary material for: Effect of social and behavioral change interventions on minimum dietary diversity among pregnant women and associated socio-economic inequality in Rajasthan, India
Source: BMC Nutr. 2024 Jun 6;10:82. doi: 10.1186/s40795-024-00887-1 (PMC11154971; doi:10.1186/s40795-024-00887-1)
Supplement: Supplementary file 2 — Supplementary Material 2 [file 40795_2024_887_MOESM2_ESM.pdf]

October 2023

Rajpusht Concurrent Monitoring (CCM) Questionnaire (राजपुष्ट समवर्ती निगरानी (सीसीएम) प्रश्नावली

महिलाओं के लिए For Women (PW, LM, MY)

Q.N.	Question	Options		Responses	Remark/Skip pattern
**SECTION 1 :**	**Respondent Identification**				
1	District (ज़िला)	5 Districts		Pre-selected	Single Choice
2	Block (खंड)			Pre-selected	Single Choice
3	Project (परियोजना)			Pre-selected	Single Choice
4	Sector (क्षेत्र)			Pre-selected	Single Choice
5	AWC (आंगनबाडी केंद्र)			Pre-selected	Single Choice
6	Type of sample (नमूना का प्रकार)	1. Cohort sample<br>2. Regular sample	1. कोहोर्ट नमूना<br>2. नियमित नमूना	Pre-selected	
7	Type of AWC's area<br>आंगनवाड़ी केंद्र के क्षेत्र का प्रकार	1. Urban<br>2. Rural<br>3. Rural-Tribal	1. शहरी<br>2. ग्रामीण<br>3. ग्रामीण-आदिवासी	Pre-selected	Single Choice
8	Name of investigator अन्वेषक का नाम				
9	Code of the investigator अन्वेषक का कोड			Pre-selected	
10	Date of survey	सर्वेक्षण की तिथि		Pre-selected	
11	Family ID परिवार आईडी	To be generated by the application	एप्लिकेशन द्वारा उत्पन्न किया जाना	Pre-selected	
12	Type of beneficiary<br>लाभार्थी का प्रकार	1. PW<br>2. LM<br>3. MY	1. PW<br>2. LM<br>3. MY	Pre-selected	
13	Age of beneficiary<br>लाभार्थी की आयु	Record in completed years	पूरे हुए वर्षों में रिकॉर्ड	Pre-selected	
14	Is PCTS ID correct?<br>क्या पीसीटीएस आईडी सही है?	1. Yes<br>2. No<br>3. Not available	1. हाँ<br>2. नहीं<br>3. उपलब्ध नहीं		
15	Result of the interview<br>साक्षात्कार का परिणाम	1. Completed<br>2. Partially Completed<br>3. Respondent not at home - went for work<br>4. Respondent not at home - went for health check-ups<br>5. Respondent temporary went to her mother place<br>6. Permanently migrated	1. पूर्ण<br>2. आंशिक रूप से पूर्ण<br>3. लाभार्थी घर पर नहीं है - काम के लिए गई है<br>4. लाभार्थी घर पर नहीं है - स्वास्थ्य जांच के लिए गई है<br>5. लाभार्थी अपनी माँ के घर (मायके) गई है<br>6. स्थायी रूप से चली गई		

CONFIDENTIAL

SECTION 2: Household Characteristics (Respondent – PW/LM/MY)		Options in English	Options in Hindi	Code	Remark
1	Sex of the head of the household<br>घर के मुखिया का लिंग	1. Male<br>2. Female	1. पुरुष<br>2. महिला	1<br>2	
2	Religion of the head of household<br>घर के मुखिया का धर्म	1. Hindu<br>2. Muslim<br>3. Christian<br>4. Sikh<br>5. Jain<br>6. Other	1. हिंदू<br>2. मुसलमान<br>3. ईसाई<br>4. सिख<br>5. जैन<br>6. अन्य	1<br>2<br>3<br>4<br>5<br>6	
3	Caste of the head of household<br>घर के मुखिया की जाति	1. Scheduled Caste<br>2. Scheduled Tribe<br>3. Other Backward Class<br>4. None of these	1. अनुसूचित जाति<br>2. अनुसूचित जनजाति<br>3. अन्य पिछड़ा वर्ग<br>4. अन्य	1<br>2<br>3<br>4	
4	What is your education completed?<br>आपकी शिक्षा का स्तर क्या है?	1. No schooling/ illiterate<br>2. Primary education (up to class 5)<br>3. Middle school (up to class 8)<br>4. High School (up to class 10)<br>5. Higher secondary (10+2)<br>6 Bachelors/ Prof degree/Masters	1. कोई स्कूली शिक्षा / अनपढ़<br>2. प्राथमिक शिक्षा (कक्षा 5 तक)<br>3. "उच्च प्राथमिक शिक्षा (कक्षा 8 तक)"<br>4. माध्यमिक (कक्षा 10 तक)<br>5. उच्च माध्यमिक (10 + 2)<br>6. बैचलर्स / प्रोफेशनल / मास्टर्स डिग्री	1<br>2<br>3<br>4<br>5<br>6	
5	How many usual members reside at your house?<br>आपके घर में सामान्यतः कितने सदस्य रहते हैं?	Members.. (Specify in number)<br>**(RANGE 0-50)**	सदस्य .... (संख्या लिखें)		
6a	House type:<br>What is the main material of the floor?<br>फर्श की मुख्य सामग्री क्या है?	1. Natural floor- Mud/Clay/Earth<br>2. Natural floor- Sand<br>3. Natural floor- Dung<br>4. Rudimentary floor- Raw wood planks<br>5. Rudimentary floor- Palm/ Bamboo<br>6. Rudimentary floor- Brick<br>7. Rudimentary floor- Stone<br>8. Finished floor- Parquet or polished wood<br>9. Finished floor- Vinyl or Ashphalt<br>10. Finished floor- Ceramic tiles	1. प्राकृतिक तल- मिट्टी/मिट्टी/ धरती<br>2. प्राकृतिक तल- रेत<br>3. प्राकृतिक तल- गोबर<br>4. अल्पविकसित तल- कच्ची लकड़ी के तख्ते<br>5. अल्पविकसित तल - ताड़/बांस<br>6. अल्पविकसित तल- ब्रिक<br>7. अल्पविकसित तल- पत्थर<br>8. तैयार मंजिल- लकड़ी की छत या पॉलिश की हुई लकड़ी	1<br>2<br>3<br>4<br>5<br>6<br>7<br>8<br>9<br>10	

CONFIDENTIAL

		11. Finished floor- Cement<br>12. Finished floor- Carpet<br>13. Finished floor- Polished stone/ Marbel/ Granite<br>14. Others (Specify)……………………………..	9. तैयार मंजिल- विनाइल या ऐशफाल्ट<br>10. तैयार मंजिल- सिरेमिक टाइलें<br>11. तैयार मंजिल- सीमेंट<br>12. तैयार मंजिल- कालीन<br>13. तैयार मंजिल- पॉलिश पत्थर / मार्बल / ग्रेनाइट<br>14. अन्य (निर्दिष्ट करें)…………………………….	11<br>12<br>13<br>14	
6b	House type:<br>What is the main material of the roof?<br><br>छत की मुख्य सामग्री क्या है?	1. Natural roofing- No roof<br>2. Natural roofing- Thatched/ Palm leaf/ Reed/ Grass<br>3. Natural roofing- Mud<br>4. Natural roofing -sod/mud and grass mixture<br>5. Natural roofing -plastic/polythene sheeting<br>6. Rudimentary roofing- Rustic mat<br>7. Rudimentary roofing- Palm/Bamboo<br>8. Rudimentary roofing- Raw Wood Planks/Timber<br>9. Rudimentary roofing- Unburnt Brick<br>10. Rudimentary roofing- Loosely Packed Stone<br>11. Finished roofing- Metal/Gi<br>12. Finished roofing- Wood<br>13. Finished roofing- Calamine/Cement Fiber<br>14. Finished roofing- Asbestos Sheets<br>15. Finished roofing- Rcc/Rbc/Cement/Concrete<br>16. Finished roofing- Roofing Shingles<br>17. Finished roofing- Tiles<br>18. Finished roofing- Slate<br>19. Finished roofing- Burnt Brick<br>20. Others (Specify)………………………………..	1. प्राकृतिक छत- कोई छत नहीं<br>2. प्राकृतिक छत- छप्पर/ताड़ का पत्ता/ रीड /घास<br>3. प्राकृतिक छत- मिट्टी<br>4. प्राकृतिक छत-सोद/मिट्टी और घास का मिश्रण<br>5. प्राकृतिक छत - प्लास्टिक / पॉलिथीन शीटिंग<br>6. अल्पविकसित छत- रस्टिक मैट<br>7. अल्पविकसित छत- ताड़/बांस<br>8. अल्पविकसित छत- कच्ची लकड़ी के तख्त/लकड़ी<br>9. अल्पविकसित छत - बिना जली ईंट<br>10. अल्पविकसित छत- ढीला-ढाला पत्थर<br>11. तैयार छत- धातु / Gi<br>12. तैयार छत- लकड़ी<br>13. तैयार छत- कैलामाइन/सीमेंट फाइबर<br>14. तैयार छत- एस्बेस्टस शीट्स<br>15. तैयार छत- आरसीसी/आरबीसी/सीमेंट/कंक्रीट<br>16. समाप्त छत- छत शिंग्लस<br>17. तैयार छत- टाइलें<br>18. तैयार छत- स्लेट<br>19. तैयार छत- जली हुई ईंट<br>20. अन्य (निर्दिष्ट करें)…………………………….	1<br>2<br>3<br>4<br>5<br>6<br>7<br>8<br>9<br>10<br>11<br>12<br>13<br>14<br>15<br>16<br>17<br>18<br>19<br>20	
6c	House type:<br>What is the main material of the exterior walls?	1. Natural walls- No Walls<br>2. Natural walls- Cane/Palm/Trunks/Bamboo<br>3. Natural walls- Mud<br>4. Natural walls- Grass/Reeds/Thatch<br>5. Rudimentary walls- Bamboo with Mud	1. प्राकृतिक दीवारें- कोई दीवार नहीं<br>2. प्राकृतिक दीवारें- बेंत/ ताड़ / तना /बांस<br>3. प्राकृतिक दीवारें- मिट्टी<br>4. प्राकृतिक दीवारें- घास / सरकंडा / छप्पर<br>5. अल्पविकसित दीवारें- मिट्टी के साथ बांस	1<br>2<br>3<br>4<br>5<br>6	

CONFIDENTIAL

	बाहरी दीवारों की मुख्य सामग्री क्या है?	6. Rudimentary walls- Stone with Mud<br>7. Rudimentary walls- Plywood<br>8. Rudimentary walls- Cardboard<br>9. Rudimentary walls- Unburnt Brick<br>10. Rudimentary walls- Raw Wood/Reused Wood<br>11. Finished Walls- Cement/Concrete<br>12. Finished walls- Stone with Lime/Cement<br>13. Finished walls- Burnt Bricks<br>14. Finished walls- Cement Blocks<br>15. Finished walls- Wood Planks/Shingles<br>16. Finished walls- GI/Metal/Asbestos Sheets<br>17. Others (Specify)…………………………………	6. अल्पविकसित दीवारें- मिट्टी के साथ पत्थर<br>7. अल्पविकसित दीवारें- प्लाईवुड<br>8. अल्पविकसित दीवारें- कार्डबोर्ड<br>9. अल्पविकसित दीवारें- बिना जली ईंट<br>10. अल्पविकसित दीवारें- कच्ची लकड़ी/पुन: उपयोग की गई लकड़ी<br>11. तैयार दीवारें- सीमेंट/कंक्रीट<br>12. तैयार दीवारें- चूना/सीमेंट के साथ पत्थर<br>13. तैयार दीवारें- जली हुई ईंटें<br>14. तैयार दीवारें- सीमेंट ब्लॉक<br>15. तैयार दीवारें- लकड़ी के तख्त / छत शिंग्लस<br>16. तैयार दीवारें- जीआई/धातु/एस्बेस्टस शीट्स<br>17. अन्य (निर्दिष्ट करें)………………………………..	7<br>8<br>9<br>10<br>11<br>12<br>13<br>14<br>15<br>16<br>17	
6d	Does any member of this household own this house or any other house?<br><br>क्या इस घर का कोई सदस्य इस घर या किसी अन्य घर का मालिक है?	1. Yes<br>2. No	1. हाँ<br>2. नहीं	1<br>2	
6e	Does any member of this household own any agricultural land?<br>क्या इस परिवार के किसी सदस्य के पास कृषि योग्य भूमि है?	1. Yes<br>2. No	1. हाँ<br>2. नहीं	1<br>2	
6f	How many rooms in this household are used for sleeping?<br>इस घर में कितने कमरे सोने के लिए उपयोग किए जाते हैं?	Rooms ………………………………….. (Specify numbers)<br><br>**RANGE 0-20**	कमरे ………………………………. (संख्या निर्दिष्ट करें)		
6g	Are there any other people who may not be members of your family such as domestic servants, lodgers or friends who usually live here?<br>क्या कोई अन्य लोग हैं जो आपके परिवार के सदस्य नहीं हो सकते हैं, आमतौर पर यहां रहते हैं?- जैसे घरेलू नौकर, रहने वाले या दोस्त	1. Yes<br>2. No	1. हाँ<br>2. नहीं	1<br>2	

CONFIDENTIAL

6h	What type of fuel does your household ***mainly*** *use* for cooking?<br>आपके परिवार में खाना बनाने के लिए मुख्य रूप से कोनसे ईंधन का प्रयोग करते है	1. Electricity<br>2. LPG/Natural Gas<br>3. Kerosene<br>4. Coal/Lignite<br>5. Charcoal/ Wood/ Dunk-Cake<br>6. Others (Specify)___________________	1. बिजली<br>2. एलपीजी / प्राकृतिक गैस<br>3. मिटटी तेल<br>4. कोयला / लिग्नाइट<br>5. लकड़ी का कोयला/ लकड़ी / डंक केक<br>6. अन्य (उल्लिखित करें) _________	1<br>2<br>3<br>4<br>5<br>6	
6i	What is the *main source* of drinking water for members of the household?<br><br>आपके घर में पीने के पानी का मुख्य स्त्रोत क्या है?	1. Piped into dwelling<br>2. Piped to yard/plot<br>3. Public tap/standpipe<br>4. Hand pump<br>5. Tube well/bore well<br>6. Protected well<br>7. Unprotected well<br>8. Tanker/truck<br>9. Cart with small tank<br>10. River/dam/lake/pond/canal/ irrigation channe<br>11. Other (specify) ___________	1. नल द्वारा<br>2. यार्ड / प्लॉट के लिए पाइप किया गया<br>3. सार्वजनिक नल / स्टैंडपाइप<br>4. हैंड पंप<br>5. नलकूप / बोरवेल<br>6. संरक्षित कुंए से<br>7. असुरक्षित कुंए से<br>8. टैंकर / ट्रक<br>9. छोटी टंकी वाली गाड़ी<br>10. नदी / बांध / झील / तालाब / नहर / सिंचाई चैनल<br>11. अन्य (उल्लिखित करें) _________	1<br>2<br>3<br>4<br>5<br>6<br>7<br>8<br>9<br>10	
6j	How do you mainly purify your drinking water before use?<br>उपयोग करने से पहले आप मुख्य रूप से अपने पीने के पानी को कैसे शुद्ध करते हैं?	1. Boil<br>2. Use Purifier<br>3. Use other methods such as (put alum/clorination/Iodine)<br>4. Do nothing	1. उबालते है<br>2. पानी साफ़ करने की मशीन इस्तेमाल करते है<br>3. फिटकरी / क्लोरीनेशन / आयोडीन/कपडे लगाने जैसे अन्य तरीकों का प्रयोग करते है<br>4. कुछ नहीं करते है	1<br>2<br>3<br><br>4	
6k	What kind of toilet facility does your household mainly use?<br>आपके घर में मुख्य रूप से किस प्रकार की शौचालय की सुविधा है?	1. Flush to Piped Sewer System / Flush Pit Latrine / Septic Tank<br>2. Improved Pit<br>3. Pit Latrine With / Without Slab<br>4. No Facility / Uses open space or field<br>5. Others (Specify)________________	1. सीवर सिस्टम में सीधे पाइप द्वारा फ्लश / फ्लश पिट लैट्रिन / सेप्टिक टैंक<br>2. सुरक्षित गड्ढे वाली टॉयलेट<br>3. छत / बिना छत वाले गड्ढेदार टॉयलेट<br>4. किसी भी प्रकार की टॉयलेट नहीं है / खुले स्थान<br>5. अन्य (उल्लिखित करें) _________	1<br><br>2<br>3<br>4<br>5	
7	Do you grow vegetables/fruits for own consumption?<br>क्या आप अपने उपभोग के लिए सब्जियां / फल उगाते हैं?	1. Yes<br>2. No	1. हाँ<br>2. नहीं	1<br>2	
8a	Does your household own any of the following animals?	1. Cows/Buffaloes<br>2. Camels<br>3. Goats<br>4. Sheep	1. गाय / भैंस<br>2. ऊंट<br>3. बकरी<br>4. भेड़	1<br>2<br>3<br>4	

CONFIDENTIAL

	क्या आपके घर में निम्नलिखित जानवरों में से कोई भी है?	5. Chickens or Ducks<br>6. None	5. मुर्गियाँ / बतखें<br>6. घर में कोई जानवर नहीं है	5<br>6	
8b	Does your household have following goods?<br><br>क्या आपके घर में निम्नलिखित सामान है ?	1. Electricity<br>2. Mattress<br>3. Pressure Cooker<br>4. Chair<br>5. Cot/Bed<br>6. Table<br>7. Electric Fan<br>8. Radio/Transistor<br>9. B & W Television<br>10. Colour Television<br>11. Sewing Machine<br>12. Mobile Telephone<br>13. Landline Telephone<br>14. Internet<br>15. Computer<br>16. Refrigerator<br>17. Air Conditioner/Cooler<br>18. Washing Machine<br>19. Watch/Clock<br>20. Bicycle<br>21. Motorcycle/Scooter<br>22. Animal-Drawn Cart<br>23. Car<br>24. Water Pump<br>25. Thresher<br>26. Tractor	1. बिजली<br>2. गद्दे<br>3. प्रेशर कुकर<br>4. कुर्सी<br>5. खाट/बिस्तर<br>6. टेबल<br>7. इलेक्ट्रिक फैन<br>8. रेडियो/ट्रांजिस्टर<br>9. ब्लैक एंड वाइट टेलीविजन<br>10. रंगीन टेलीविजन<br>11. सिलाई मशीन<br>12. मोबाइल टेलीफोन<br>13. लैंडलाइन टेलीफोन<br>14. इंटरनेट<br>15. कंप्यूटर<br>16. फ्रिज<br>17. एयर कंडीशनर/कूलर<br>18. वॉशिंग मशीन<br>19. हाथ वाली घडी/ दीवाल वाली घडी<br>20. साइकिल<br>21. मोटरसाइकिल/स्कूटर<br>22. पशु- कार्ट<br>23. कार<br>24. पानी पंप<br>25. थ्रेशर<br>26. ट्रैक्टर	1<br>2<br>3<br>4<br>5<br>6<br>7<br>8<br>9<br>10<br>11<br>12<br>13<br>14<br>15<br>16<br>17<br>18<br>19<br>20<br>21<br>22<br>23<br>24<br>25<br>26	
8c	Does any usual member of this household have a bank account or a post office account?<br>क्या इस घर के किसी सदस्य का बैंक खाता या डाकघर खाता है?	1. Yes<br>2. No<br>3. Don't know	1. हाँ<br>2. नहीं<br>3. पता नहीं	1<br>2<br>3	
9	Does this household have a NREGA card?	a. Yes<br>b. No	1. हाँ<br>2. नहीं	1<br>2	

CONFIDENTIAL

10	What type of ration card does your household have?<br>आपके घर में किस प्रकार का राशन कार्ड है?	1. APL (Blue/ green Card)<br>2. BPL (Dark pink)<br>3. Antodya (Yellow)<br>4. General (White card)<br>5. Does not have any card	1. एपीएल (नीला / हरा कार्ड)<br>2. बीपीएल (गहरा गुलाबी)<br>3. अंत्योदय (पीला)<br>4. सामान्य (सफेद कार्ड)<br>5. कोई कार्ड नहीं है	1<br>2<br>3<br>4<br>5	

Women's lifestyle and Morbidity status

S.NO	Question			
12	A. Do you currently have any of these diseases?<br>क्या आपको इस समय इनमें से कोई रोग है?	Options	B .**If yes,** have you sought treatment for this problem?<br>यदि हां, क्या आपने इसका इलाज करवाया?	Options
	Diabetes?<br>मधुमेह [डायिबटीज]	Yes<br>No<br>Don't Know	Diabetes?<br>मधुमेह [डायिबटीज]	Yes<br>No
	Hypertension?<br>उच्च रक्तचाप (बीपी)	Yes<br>No<br>Don't Know	Hypertension?<br>उच्च रक्तचाप (बीपी)	Yes<br>No
	A chronic respiratory disease including asthma?<br>दमा [अस्थमा] /पुरानी साँस संबंधी रोग	Yes<br>No<br>Don't Know	A chronic respiratory disease including asthma?<br>दमा [अस्थमा] /पुरानी साँस संबंधी रोग	Yes<br>No
	Goitre or any other thyroid disorder?<br>गलगण्ड या अन्य थाइराइड संबंधी विकार [रोग]	Yes<br>No<br>Don't Know	Goitre or any other thyroid disorder?<br>गलगण्ड या अन्य थाइराइड संबंधी विकार [रोग]	Yes<br>No
13	Do you currently smoke cigarettes every day, some days, or not at all?<br>क्या आप वर्तमान में हर दिन सिगरेट पीते हैं, कुछ दिन, या बिल्कुल नहीं?		EVERY DAY<br>SOME DAYS<br>NOT AT ALL<br><br>हर दिन<br>किसी दिन<br>बिल्कुल भी नहीं	
14	On average, how many cigarettes do you currently smoke each day?<br>वर्तमान में आप प्रतिदिन औसतन कितनी सिगरेट पीते हैं?		Number……<br><br>**RANGE 0-50**	

CONFIDENTIAL

15	For how long have you been smoking cigarettes regularly?<br><br>आप कितने समय से नियमित रूप से सिगरेट पी रहे हैं?	IF LESS THAN 1 MONTH, RECORD WEEKS – Weeks ----<br>IF LESS THAN 2 YEARS, RECORD MONTHS- Months ----<br>IF 2 OR MORE YEARS, RECORD YEARS- Years ----<br>Never Smoked Regularly ---
16	Do you currently smoke bidis every day, some days, or not at all?<br>क्या आप वर्तमान में हर दिन बीड़ी पीते हैं, कुछ दिन, या बिल्कुल नहीं?	EVERY DAY<br>SOME DAYS<br>NOT AT ALL<br><br>हर दिन<br>किसी दिन<br>बिल्कुल भी नहीं
17	Do you currently chew tobacco everyday, some day, or not at all?<br>क्या आप वर्तमान में हर दिन बीड़ी पीते हैं, कुछ दिन, या बिल्कुल नहीं?	EVERY DAY<br>SOME DAYS<br>NOT AT ALL<br><br>हर दिन
18	Do you drink alcohol?<br>क्या आप शराब पीती हैं?	Yes<br>No<br>1. हाँ<br>2. नहीं
19	How often do you drink alcohol: almost every day, about once a week or less than once a week? आप कितनी बार शराब पीते हैं: लगभग हर दिन, सप्ताह में लगभग एक बार या सप्ताह में एक बार से कम?	Almost everyday<br>About once a week<br>Less than once a week<br><br>लगभग हर दिन<br>लगभग एक सप्ताह में एक बार सप्ताह में एक बार से भी कम

CONFIDENTIAL

SECTION 3 : Service Assessment of PW		Options in English	Options in Hindi	Code	Remark
1	Category of beneficiary<br>लाभार्थी की स्थिति<br>(Pre-selected from PC App)	1. Pregnant woman (PW)<br>2. Lactating mother (LM) of < 6 months child<br>3. Mother of Young Child (MY) of 6-36 months child	1. गर्भवती महिला (पीडब्ल्यू)<br>2. स्तनपान कराने वाली < 6 माह के छोटे बच्चों की मां (एलएम)<br>3. 6-36 माह के छोटे बच्चों की मां (एलएम)		
2	PMMVY/IGMPY beneficiary<br>PMMVY/IGMPY लाभार्थी<br>(Pre-selected from PC App)	1. Parity 1 – 1st time pregnant or LM/MY with one child<br>2. Parity 2 - 2nd time pregnant or LM/MY with two children	1. पहली बार गर्भवती या एक बच्चे के साथ एलएम / एमई<br>2. दूसरी बार गर्भवती या दो बच्चे के साथ एलएम / एमई	(Pre-selected)	
3	How many months of pregnancy are you in currently?<br>वर्तमान में आप कितने महीनों की गर्भावस्था में हैं?<br>(Pre-selected from PC App)	Numeric (up to 9)	संख्यात्मक (9 तक)		
4	When pregnancy was registered at the AWC?<br>AWC में अपनी गर्भावस्था को पंजीकृत किया था तब आप कितने महीने की गर्भवती थीं?<br>(Record from Mamta card)	1. Date of registration<br>2. Did not register	1. रजिस्टर<br>2. रजिस्टर नहीं किया		
	**Ask Mamta card and record the following and record actual number**				
5a	Is Mamta card available?<br>क्या ममता कार्ड उपलब्ध है?	1. Mamta card available<br>2. Mamta card available but not updated<br>3. Mamta card not available	1. ममता कार्ड उपलब्ध<br>2. ममता कार्ड उपलब्ध लेकिन अपडेट नहीं<br>3. ममता कार्ड उपलब्ध नहीं है	1<br>2<br>3	
6a	Hb level and date<br>एचबी स्तर और तारीख<br>(Record from Mamta card)	1. Last Hb level with one decimal point<br>2. Date of last Hb measured	1. अंतिम एचबी स्तर एक दशमलव बिंदु के साथ<br>2. अंतिम एचबी मापी जाने की तिथि	1<br>2	
6b	How many ANCs completed so far? (information and date)<br>अब तक कितने एएनसी पूरे हुए हैं? (सूचना और तारीख)?	1. Number of ANCs<br>2. Date of last ANC	1. एएनसी की संख्या<br>2. अंतिम एएनसी की तिथि	1<br>2	

CONFIDENTIAL

7	During your last ANC did you receive the following services?<br><br>आपके अंतिम ANC के दौरान, आपको निम्नलिखित में से कौन सी सेवा प्राप्त हुई?	a. Weight taken<br>b. Counselling on diet and nutrition<br>c. Blood pressure measured<br>d. Hb test done<br>e. Sugar test done<br>f. Urine tested<br>g. Abdominal check-up done<br>h. IFA supplements given<br>i. Calcium Tablets given<br>j. TT Injection given<br>k. Deworming medication given	a. वजन लिया गया<br>b. आहार और पोषण पर परामर्श<br>c. रक्तचाप (BP) को मापा गया<br>d. एचबी परीक्षण किया<br>e. शुगर टेस्ट किया<br>f. पेशाब की जांच<br>g. पेट की जाँच की<br>h. IFA/Iron की खुराक दी<br>i. कैल्शियम की गोलियां दी<br>j. टीटी इंजेक्शन दिया<br>k. पेट के कीड़े मारने वाली दवाई	a<br>b<br>c<br>d<br>e<br>f<br>g<br>h<br>i<br>j<br>k	
8	How many IFA tablets did you receive/procure in last one month?<br>पिछले एक महीने में आपको कितने IFA टैबलेट मिले / खरीदे गए?	No. of IFA Tablets<br><br>**(RANGE 0-100)**	आईएफए गोलियों की संख्या<br><br>(रेंज 0-60)	Numeric	
9	How many IFA Tablets did you consume in last one month?<br>पिछले एक महीने में आपने कितने IFA टैबलेट का का सेवन किया?	No. of IFA Tablets<br><br>**(RANGE 0-100)**	आईएफए गोलियों की संख्या<br><br>(रेंज 0-60)	Numeric	
10a	How many calcium tablets did you receive/procure in last one month?<br>पिछले एक महीने में आपको कितनी कैल्शियम की गोलियां मिली / प्राप्त हुई?	No. of Calcium Tablets<br><br>**(RANGE 0-100)**	कैल्शियम गोलियों की संख्या<br><br>(रेंज 0-60)	Numeric	
10b	How many calcium Tablets did you consume in last one month?<br>पिछले एक महीने में आपने कितने कैल्शियम टैबलेट का सेवन किया?	No. of Calcium Tablets<br><br>**(RANGE 0-60)**	कैल्शियम गोलियों की संख्या<br><br>(रेंज 0-60)	Numeric	
11a	In the last one month did you receive the THR for the entire month from the AWC?<br>क्या आपको पिछले एक महीने में आंगनवाड़ी केंद्र से पूरे महीने का टीएचआर मिला है?	1. Yes<br>2. No<br>3.	1. हाँ<br>2. नहीं<br>3. आंशिक रूप से	1<br>2<br>3	If code is 3, go to Q 12

CONFIDENTIAL

11b	***If yes or Partially received, in 11a*** In the last month what THR you received from the AWC?<br>यदि आंशिक रूप से, पिछले महीने में, आपने AWC से कितना टीएचआर प्राप्त किया?	a) Wheat<br>b) Rice<br>c) Chana dal<br>d) Other________ Specify<br>e) Not received THR from AWC	a) गेहूं<br>b) चावल<br>c) चना दाल<br>d) अन्य________ निर्दिष्ट करें<br>e) 5. आंगनवाड़ी केंद्र से टीएचआर प्राप्त नहीं हुआ		
11c	**If LM;** Did you received THR for yourself?<br>यदि LM, क्या आपने अपने लिए THR प्राप्त किया?	1. Yes<br>2. No	1. हाँ<br>2. नहीं	1<br>2	
11d	**If MY;** Did you received THR for your child?<br>यदि MY, क्या आपने अपने बच्चे के लिए THR प्राप्त किया?	1. Yes<br>2. No	1. हाँ<br>2. नहीं		
12	***If no or partially received THR,*** what was the main reason?<br><br>यदि नहीं या आंशिक रूप से THR प्राप्त हुआ तो मुख्य कारण क्या था?	1. THR was not available at the AWC<br>2. AWC did not open<br>3. Self-opt out (khud se chhod diya)<br>4. THR quality was poor<br>5. AWC was far away<br>6. Not allowed by the family<br>7. AWW did not provide me THR for personal reasons<br>8. Inadequate quantity available in the AWC<br>9. Others (specify)	1. AWC में THR (Ration) / पोषाहार उपलब्ध नहीं था<br>2. AWC नहीं खोला<br>3. सेल्फ ऑप्ट आउट/ खुद से छोड़ दिया<br>4. THR (Ration) / पोषाहार की गुणवत्ता खराब थी<br>5. AWC दूर था<br>6. परिवार द्वारा अनुमति नहीं है<br>7. AWW ने मुझे व्यक्तिगत कारणों से टीएचआर प्रदान नहीं किया<br>8. AWC में उपलब्ध अपर्याप्त मात्रा<br>9. अन्य (उल्लिखित करें)	1<br>2<br>3<br>4<br>5<br>6<br>7<br>8<br>9	
13	How did you use the THR mainly?<br><br>आपने मुख्य रूप से THR का उपयोग कैसे किया?	1. Consumed fully by self<br>2. Consumed by all family members<br>3. Gave it away to others/neighbours<br>Sold in market<br>4. Gave it to the animals/discarded/dumped<br>5. Did not use THR at all (still lying unused)<br>6. Others (specify)	1. पूरी तरह से स्वयं द्वारा सेवन किया गया<br>2. परिवार के सभी सदस्यों द्वारा सेवन किया गया<br>3. इसे दूसरों/पड़ोसियों को दे दो<br>बाजार में बिकता है<br>4. इसे जानवरों को दे दिया / फेंक दिया<br>5. टीएचआर का बिल्कुल भी उपयोग नहीं किया (अभी भी अप्रयुक्त पड़ा हुआ है)<br>6. अन्य (निर्दिष्ट करें)	1<br>2<br>3<br>4<br>5<br>6	
14	How many times you took meals yesterday?	No. of times - Meals<br>**(RANGE 0-15)**			

CONFIDENTIAL

15. Did you consume following food items (Ask one by one all food items and record as told by women for yesterday and times in a week)

क्या आपने निम्नलिखित खाद्य पदार्थों का सेवन किया (एक-एक करके सभी खाद्य पदार्थ पूछें और महिलाओं द्वारा बताए गए कल और सप्ताह में समय के अनुसार रिकॉर्ड करें)

SN	*Ask one by one all food items and record as told by women for yesterday and times in a week*		**Consumed Yesterday**		**Consumed how many days in a week**	SN	*Ask one by one all food items and record as told by women for yesterday and times in a week*		**Consumed Yesterday**		**Consumed how many days in a week**
	कल क्या क्या खाया, सप्ताह में		**कल क्या क्या खाया?**		**सप्ताह में कितने दिन खाया?**		***कल क्या क्या खाया, सप्ताह में***		**कल क्या क्या खाया?**		**सप्ताह में कितने दिन खाया?**
	कितनी बार के बारे में पूछें और रिकॉर्ड करें						***कितनी बार के बारे में पूछें और रिकॉर्ड करें***				**(RANGE 0-7)**
					**(RANGE 0-7)**						
1	**CEREALS**	**अनाज**	**Yes हाँ**	No	Number	5	**VITAMIN A RICH FRUITS**	**विटमिन ए रिच फ्रूट्स**	**Yes हाँ**	No	Number
1a	Atta/Suji ka Halwa	अट्टा / सूजी का हलवा	1	2	Number	5a	Ripe mango (fresh or dried)	पके आम (ताजा या सूखा)	1	2	Number
1b	Corn	**मक्का**	1	2	Number	5b	Ripe papaya	पका पपीता	1	2	Number
1c	Wheat	**गेहू**	0	2	Number	5c	Pumpkin	कद्दू	1	2	Number
1d	Daliya / Multi grain-based porridge	दलिया / बहु अनाज आधारित दलिया	1	2	Number	5d	Carrots	गाजर	1	2	Number
1e	Khichadi	खिचड़ी	1	2	Number	5e	Sweet potatoes that are yellow or orange inside	मीठे आलू जो अंदर पीले या नारंगी होते हैं	1	2	Number
1f	Paratha	पराठा	1	2	Number	5f	Tomato	टमाटर			

CONFIDENTIAL

1g	Roti	रोटी	1	2	Number	5g	Carrot	गाजर			
1h	Dhokla (Besan)	ढोकला (बेसन)	1	2	Number	5h	Sweet Potato	शकरकंद			
1i	Murmura	मुरमुरा	1	2	Number	5i	Cholaai ki patta	चोलाई का पत्ता			
1j	Upma (Suji)	उपमा (सूजी)	1	2	Number	5j	Sahajan ka patta	सहजन का पत्ता			
1k	Poha (Rice)	पोहा (चावल)	1	2	Number	5k	Kharbuja	खरबूजा			
1l	Bati	**बाटी**	1	2	Number	5l	Timrufal	टिमरुफल			
1m	Churma	चूरमा	1	2	Number	6	**OTHER FRUITS**	**अन्य फल**			
1n	Chawal/Rice	चावल	1	2	Number	6a	Ber	**बेर**	1	2	Number
1o	Bajra	बाजरे	1	2	Number	6b	Bel	बेल	1	2	Number
1p	Jowar	ज्वार	1	2	Number	6c	Apple	सेब	1	2	Number
1q	Guli ki Rabadi (Barley and Butter Milk)	गुल्ली की रबाड़ी (जौ और मक्खन दूध)	1	2	Number	6d	Banana	केला	1	2	Number
1r	Chila	चिला	1	2	Number	6e	Water melon	तरबूज	1	2	Number
1s	Patharia	पथरिया	1	2	Number	6f	Sitapal	सीताफल	1	2	Number
1t	Coloma	कोलोमा	1	2	Number	6g	Guava	अमरूद	1	2	Number
1u	Kali Kamod	काली कमोद	1	2	Number	6h	Grape	अंगूर	1	2	Number
1v	Kodra	कोड्रा	1	2	Number	6i	Dates	खजूर	1	2	Number
1w	Batwa/Mal	बटवा/माल	1	2	Number	6j	Chiku	चीकू	1	2	Number
1x	China	चीना	1	2	Number	6k	Jamun	जामुन	1	2	Number
1y	Kang	कांग	1	2	Number	6l	Pine apple	अनानास	1	2	Number
2	**ROOTS AND TUBERS**	**जड़ें और ट्यूबर (जमीन के अंदर पैदा होने वाली सब्जियां)**				6m	Singara	सिंगारा	1	2	Number
2a	Potatoes	आलू	1	2	Number	6k	Baharikand	बहरीकांड	1	2	Number
2b	Arabi	अरबी	1	2	Number	6l	Bandarbati	बन्दरबाटी	1	2	Number
2c	Jaami kand	**जिमीकंद**	1	2	Number	6m	Tamarind	इमली	1	2	Number
2d	Beet root or any other foods made from roots	चुकंदर या जड़ों से बने अन्य खाद्य पदार्थ	1	2	Number	6n	Raisin	किशमिश	1	2	Number
						6O	Pomegranate	अनार	1	2	Number

CONFIDENTIAL

3	**DARK GREEN LEAFY VEGETABLES**	**गहरा हरे पत्ते वाली सब्जियां**				7	**MEAT AND FISH**	**मांस/ मछली/अंडे**			
3a	Spinach	पालक	1	2	Number	7a	Beef	**बड़े जानवर का मॉस**	1	2	Number
3b	Drumstick leaves	**सहजन के पत्ते**	1	2	Number	7b	Goat	बकरा	1	2	Number
3c	Coriander leaves	धनिए के पत्ते	1	2	Number	7c	Chicken / Duck	चिकन	1	2	Number
3d	Fenugreek leaves	कसूरी मेथी	1	2	Number	7d	**EGGS**	**अंडे**	1	2	
3e	Pudina	पुदीना	1	2	Number						
3f	Cauliflower leaves	फूलगोभी के पत्ते	1	2	Number	8	**LEGUMES, NUTS AND SEEDS**	**दाल , NUTS और बीज**			
3g	Beet root leaves	चुकंदर की पत्तियां	1	2	Number	8a	Arhar dal	अरहर की दाल	1	2	Number
3h	Carrot leaves	गाजर के पत्ते	1	2	Number	8b	Chana dal	चना दाल	1	2	Number
3i	Radish leaves	मूली के पत्ते	1	2	Number	8c	Moong dal	मूंग की दाल	1	2	Number
3j	Onion stalk	प्याज का डंठल	1	2	Number	8d	Moth	मोठ	1	2	Number
3k	Mustard leaves	सरसों के पत्ते	1	2	Number	8e	Masoor dal	मसूर की दाल	1	2	Number
3l	Bathua	बथुआ	1	2	Number	8f	Urad dal	उड़द दाल	1	2	Number
3m	Cabbage	पता गोभी	1	2	Number	8g	Rajma	राजमा	1	2	Number
3n	Cauliflower leaves	फूलगोभी के पत्ते	1	2	Number	8h	Soyabean	सोया बीन	1	2	Number
3o	Garmela	गार्मेला	1	2	Number	8i	Moongfali	मूंगफली	1	2	Number
3p	Bokna	बोकना	1	2	Number	8j	Kala Chana	काला चना	1	2	Number
3q	Kachnar	कचनार	1	2	Number	8k	Chola	**छोले**	1	2	Change as suggested
3r	Dhimari	धिमारी	1	2	Number	8l	Lobia	**लोबिया**	1	2	Change as suggested
3s	Rajan	राजन	1	2	Number	8m	Matar	मटर	1	2	Number

4	**OTHER VEGETABLES**	**अन्य सब्जियां**				8n	Kulti Dal	कुल्टी दाल	1	2	Number
4a	Bitter gourd	करेला	1	2	Number	8o	Gajak – groundnut	गजक - मूंगफली	1	2	Number
4b	Lauki	लौकी	1	2	Number	8p	Til	**तिल**	1	2	Number
4d	Cauliflower	पत्ता गोभी	1	2	Number	8q	Gadda ki sabaji	**गट्टे की सब्जी**	1	2	Number
4e	Parwal	परवल	1	2	Number	8r	Pappad ki sabaji	पापड़ की सब्जी	1	2	Number
4f	Cucumber	खीरा	1	2	Number	8s	Chalar	चलर	1	2	Number
4g	Onion	प्याज	1	2	Number	8t	Saboot arhar	साबुत अरहर	1	2	Number
4h	Brinjal	बैंगन	1	2	Number	8u	Lobiya	लोबिया	1	2	Number
4i	Ladiesfinger	भिन्डी	1	2	Number	8v	Pesta	पेस्टा	1	2	Number
4j	Beans-cluster	**गँवार फली**	1	2	Number	8w	Almond	बादाम	1	2	Number
4k	French beans	फ्रेंच बीन्स	1	2	Number	8x	Cashew	काजू	1	2	Number
4l	Cauliflower	गोभी	1	2	Number	8z	Coconut	नारियल	1	2	Number
4m	Drumstick	सहजनफली	1	2	Number	9	**MILK AND MILK PRODUCTS**	**दूध और दूध उत्पाद**			
4n	Drumstick flower	**सहजन**	1	2	Number	9a	Paneer	पनीर	1	2	Number
4o	Jack fruit	**कटहल**	1	2	Number	9b	Curd	दही	1	2	Number
4p	Ladies finger	भिन्डी	1	2	Number	9c	Shrikant	श्रीखंड	1	2	Number
4q	Pumpkin	कद्दू	1	2	Number	9d	Rabadi	**राबड़ी**	1	2	Number
4r	Ridge gourd	तोरई	1	2	Number	9e	Butter milk	छाछ	1	2	Number
4s	Snake gourd	चिचिण्डा	1	2	Number	9f	Milk or other milk product	दूध या अन्य दुग्ध उत्पाद	1	2	Number
4t	Tinda	टिंडा	1	2	Number	9g	Raita	रायता	1	2	Number
~~4u~~	~~Tomatoes~~	~~टमाटर~~	~~1~~	~~2~~	~~Number~~						
4v	Ker/kair sangiri	केर / केर संगीरी	1	2	Number	10	**OILS AND FATS**				
4w	Kachar/kachri	कछार / कचहरी	1	2	Number	10a	Ghee	घी	1	2	Number
4x	Peas	मटर	1	2	Number	10b	Kachi Ghani oil	कच्ची घानी का तेल	1	2	Number

CONFIDENTIAL

4y	Unriped banana	कच्चा केला	1	2	Number	10c	Butter added to food or used for cooking	मक्खन भोजन में जोड़ा जाता है या खाना पकाने के लिए उपयोग किया जाता है	1	2	Number
4z	Mushroom	मशरूम	1	2	Number	11	**SWEETS**	**मिठाइयाँ**	1	2	
4aa	Aloe-vera	एलोविरा	1	2	Number	11a	Sugar	**चीनी से बनी मिठाई**	1	2	Number
4ab	Lotus stem	कमल गट्टा	1	2	Number	11b	Jaggery	**गुड़ से बनी मिठाई**	1	2	Number
4ac	Balam kakri	बालम काकरी	1	2	Number	11c	Honey	**शहद से बनी मिठाई**	1	2	Number
4ad	Kakdi	काकड़ी	1	2	Number	11d	Sweetened juice drinks	**मीठा जूस**	1	2	Number
4ae	Chilli	मिर्च	1	2	Number	11e	Chocolates	चॉकलेट	1	2	Number
4af	Tinda	टिंडा	1	2	Number	11f	Candies	कैंडी	1	2	Number
4ag	Lemon	नींबू	1	2	Number	11g	Cookies	कुकीज़	1	2	Number
4ah	Arbi	अरबी	1	2	Number	11h	Cakes	केक	1	2	Number
4ai	Bhambar Laat	भांबर लाट	1	2	Number						
4aj	Sem fali	सेम फली	1	2	Number	12	**BEVERAGES**	**पेय पदार्थ**	1	2	
4ak	Amla	अमला	1	2	Number	12a	Coffee	कॉफ़ी	1	2	Number
4al	Wakla	वकला	1	2	Number	12b	Tea	चाय	1	2	Number
4ai	Onion	प्याज	1	2	Number	12c	Alcoholic beverages	मादक पेय			
4aj	Raddish	मूली	1	2	Number						

CONFIDENTIAL

16a	If yes in Q15 how many bowl (katori) DARK **GREEN LEAFY VEGETABLES** you eat yesterday?		यदि हाँ, तो कल कितने कटोरी गहरे हरे पत्ते वाली सब्जियां खाये	Number	
16b	If yes in Q15 how many bowl (katori) **DAL (LEGUMES NUTS AND SEEDS)** you eat yesterday?		यदि हाँ, तो कल आप कितने कल कितने कटोरी दाल खाये?	Number	
16c	If yes in Q15 how much **VITAMIN A RICH FRUITS** (in grams) you eat yesterday?		यदि हाँ, तो कल आप कितने फल (ग्राम में) खाये ?	In grams	
16d	If yes in Q15 how much **MILK AND MILK PRODUCTS** (in Liter) you took yesterday?		यदि हाँ, तो कल आपने कल दूध या दूध से बने उत्पाद कितना (ग्राम में) लिया?	In Liter	
16e	If yes in Q15 how many bowls **meet/fish/ EGGS** you eat yesterday?		यदि हाँ, तो कल आप कितने कटोरी मांस/ मछली/अंडे खाये?	Number	
17	How many times between the meals you took Snacks (अल्प आहार)? Yesterday? कल आपने भोजन के बीच कितनी बार नाश्ता किया?	Number of times eat Snacks yesterday<br>**(RANGE 0-10)**	कल कितनी बार नाश्ता किया	Number	
18	If 1 or more in Q17 what snack did you eat yesterday?	a) Local fruit /juice<br>b) Sweet potato<br>c) Milk and milk products<br>d) Rabadi<br>e) Raab/ Rabadi<br>f) Lassi/ Chhach<br>g) Gur and Moongfali<br>h) Peanuts<br>i) Chikki<br>j) Chiwada/Murmura/Bhelpuri<br>k) Halwa<br>l) Ladoo<br>m) Upma<br>n) Poha<br>o) Daliya<br>p) Biscuit	a. स्थानीय फल/फलों का रस<br>b. शकरकंद<br>c. दूध या दूध से बने पदार्थ / दही<br>d. रबाड़ी<br>e. राब / रबाड़ी<br>f. लस्सी / छाछ<br>g. गुड़ मूंगफली<br>h. मूंगफली<br>i. चिकी<br>j. चिवड़ा / मुरमुरा / भेलपुरी<br>k. हलवा (मूंगदाल, बेसन, सूजी, आटा या बहु अनाज)	a<br>b<br>c<br>d<br>e<br>f<br>g<br>h<br>i<br>j<br>k<br>l<br>m<br>n<br>o<br>p	Multiple choice

CONFIDENTIAL

		q) Samosa/ Pokada<br>r) Matri<br>s) Other- Maggie, Kurkure/Tanatan/cold drinks/ Icecream/soda/ etc	l. लड्डू -(तिल, बेसन, सूजी, आटा या बहु अनाज)<br>m. उपमा<br>n. पोहा<br>o. दलिया<br>p. बिस्कुट<br>q. समोसा/पकोड़ा<br>r. मठरी<br><br>s. अन्य- मैगी, कुरकुरे/टानाटन/कोल्ड ड्रिंक्स/आइसक्रीम/सोडा)<br>a.		
19	How many bowls (katori) snacks you eat yesterday?<br>आपने कल कितने कटोरी नाश्ता किया था?	Number<br>**(RANGE 0-10)**	Number	Number	

CONFIDENTIAL

Section 4: Exposure to Mass Media and SBCC (PW LM MY)		Options in English	Options in Hindi	Code	Remark
	**Mass media questions:**				
1	Did you watch/ listen/ read following the following in last three months?<br>क्या आपने पिछले तीन महीनों में निम्नलिखित को देखा / पढ़ा / सुना है?	a. TV<br>b. Radio<br>c. Newspaper<br>d. None of the above	a. टीवी<br>b. रेडियो<br>c. समाचार पत्र<br>d. इनमे से कोई भी नहीं	a<br>b<br>c<br>d	
2a	If TV, 1 , Have you come across any nutrition related advertisement/ films/ videos/ songs during the last three months on TV?<br>क्या आपने टीवी पर पिछले तीन महीनों के दौरान पोषण संबंधी कोई विज्ञापन/फिल्में/वीडियो/गीत देखे हैं?	1. Yes<br>2. No	1. हाँ<br>2. नहीं	1<br>2	
2b	***If yes, 2a,*** what do you recall about nutrition related messages on TV in last three months?<br>यदि हाँ, तो आपको पिछले तीन महीनों में टीवी पर पोषण संबंधी संदेशों के बारे में क्या याद है?	a. About cash transfer schemes for nutrition<br>b. About nutrition during pregnancy<br>c. About different foods that are taken during pregnancy<br>d. Not remember any message	a. गर्भावस्था से संबंधित नकद हस्तांतरण योजनाओं के बारे में<br>b. गर्भावस्था के दौरान पोषण के बारे में<br>c. गर्भावस्था के दौरान लिए जाने वाले विभिन्न खाद्य पदार्थों के बारे में<br>d. याद नहीं है	a<br>b<br>c<br>d	
3a	***If Newspaper in 1,*** Have you come across any nutrition related advertisement/ message during the last three months on Newspaper?<br>यदि समाचार पत्र पढ़ा तो क्या पिछले तीन महीनों के दौरान कोई पोषण संबंधी विज्ञापन / संदेश पढ़ा है?	1. Yes<br>2. No	1. हाँ<br>2. नहीं	1<br>2	
3b	***If yes in 3a,*** what do you recall about nutrition related messages in a newspaper in last three months?<br>यदि हाँ, तो आपको पिछले तीन महीनों में समाचार पत्रों से पोषण संबंधी संदेशों के बारे में क्या याद है?	a. About cash transfer schemes for nutrition<br>b. About nutrition during pregnancy<br>c. About different foods that are taken during pregnancy<br>d. Not remember any message	a. गर्भावस्था से संबंधित नकद हस्तांतरण योजनाओं के बारे में<br>b. गर्भावस्था के दौरान पोषण के बारे में<br>c. गर्भावस्था के दौरान लिए जाने वाले विभिन्न खाद्य पदार्थों के बारे में<br>d. याद नहीं है	a<br>b<br>c<br>d	
4a	***If Radio in 1,*** Have you come across any nutrition related advertisement/ songs during the last three months on Radio?<br>क्या आपने पिछले तीन महीनों के दौरान रेडियो पर पोषण संबंधी कोई विज्ञापन/गीत देखा है?	1. Yes<br>2. No	1. हाँ<br>2. नहीं	1<br>2	

CONFIDENTIAL

4b	If yes in 4a, what do you recall about nutrition related messages on radio in last three months?<br>आपको पिछले तीन महीनों में रेडियो पर पोषण संबंधी संदेशों के बारे में क्या याद है?	About cash transfer schemes for nutrition<br>About nutrition during pregnancy<br>About different foods that are taken during pregnancy<br>Not remember any message	गर्भावस्था से संबंधित नकद हस्तांतरण योजनाओं के बारे में<br>गर्भावस्था के दौरान पोषण के बारे में<br>गर्भावस्था के दौरान लिए जाने वाले विभिन्न खाद्य पदार्थों के बारे में<br>याद नहीं है	a<br>b<br>c<br>d	

CONFIDENTIAL

	Social media question				
5a	Did you use any of the following social media platform in last three months?<br>क्या आपने पिछले तीन महीनों में किसी भी सोशल मीडिया (फेसबुक, यूट्यूब, WhatsApp etc.) का उपयोग किया है?	a. Facebook<br>b. Youtube<br>c. WhatsApp<br>d. Moj<br>e. Takatak<br>f. Instagram<br>g. Any other (such as….)<br>h. Not using any above media	a. फेसबुक<br>b. यूट्यूब<br>c. WhatsApp<br>d. मोज,<br>e. टका टक<br>f. इंस्टाग्राम<br>g. अन्य कोई जैसे<br>h. उपरोक्त का उपयोग नहीं कर रहा है	a<br>b<br>c<br>d<br>e	If not using any social media (code e) then go to Q6a
5b	Have you come across any nutrition related advertisement/ films/ videos/ songs during the last three months on any social media (Facebook/Youtube/WhatsApp)?<br><br>क्या आपने पिछले तीन महीनों के दौरान किसी भी सोशल मीडिया (फेसबुक / यूट्यूब / व्हाट्सएप) पर किसी भी पोषण संबंधी विज्ञापन / फिल्मों / वीडियो / गीतों को देखा है?	1. Yes<br>2. No	1. हाँ<br>2. नहीं	1<br>2	
5c	***Ask without showing different items***<br><br>***If Yes, in 5b,*** Have you come across any nutrition related advertisement/ films/ videos/ songs on social media during the last three months?<br><br>क्या आप पिछले तीन महीनों के दौरान सोशल मीडिया पर किसी भी पोषण संबंधी विज्ञापन / फिल्मों / वीडियो / गीतों को लेकर आए हैं	a. About gaining weight during pregnancy<br>b. About nutrition for pregnant women<br>c. About what to eat during pregnancy<br>d. About Colostrum feeding<br>e. About exclusive breastfeeding<br>f. Family responsibility for child feeding<br>g. About husband's roles and responsibility during pregnancy<br>h. Family planning<br>i. Complementary feeding<br>j. About cash transfer (PMMVY/IGMPY)<br>k. Any other	a. गर्भावस्था के दौरान वजन बढ़ने के बारे में<br>b. गर्भवती महिलाओं के पोषण के बारे में<br>c. गर्भावस्था के दौरान क्या खाएं<br>d. कोलोस्ट्रम (पीला गाढ़ा दूध) के बारे में<br>e. स्तनपान के बारे में<br>f. बच्चे को खिलाने के लिए पारिवारिक जिम्मेदारी<br>g. गर्भावस्था के दौरान पति की भूमिका और जिम्मेदारी के बारे में<br>h. परिवार नियोजन<br>i. पूरक भोजन<br>j. नकद हस्तांतरण के बारे में (PMMVY/IGMPY)<br>k. अन्य कोई	a<br>b<br>c<br>d<br>e<br>f<br>g<br>h<br>i<br>j<br>k	

CONFIDENTIAL

	Mid Media Exposure				
6a	Have you seen wall paintings in your area (AWC/ School/ Panchayat/ SC/ PHC etc.) in last three months?<br><br>क्या आपने पिछले तीन महीनों में अपने क्षेत्र (आंगनवाड़ी केंद्र / स्कूल / पंचायत / चिकित्सा केंद्र / प्राथमिक स्वास्थ्य केंद्र आदि) में दीवार पेंटिंग देखी है?	1. Yes<br>2. No	1. हाँ<br>2. नहीं	1<br>2	
6b	***If Yes, in 6a***, What do you recall about the themes of these wall painting?<br><br>यदि हां, आपको इन वॉल पेंटिंग के विषयों के बारे में क्या याद है?	a. Swachch bharat abhiyaan<br>b. Maternal nutrition/poshan abhiyaan<br>c. Government schemes<br>d. Any other	a. स्वच्छ भारत अभियान<br>b. मातृ पोषण / पोषण अभियान<br>c. अन्य सरकारी योजनाएं<br>d. अन्य कोई	a<br>b<br>c<br>d	
7a	Have you seen wall hanging posters in the anganwadi centers or Gram Panchayat building/Sub-Centre in last three months?<br><br>क्या आपने पिछले तीन महीनों में आंगनवाड़ी केंद्रों में दीवार पर लटकने वाली पोस्टर देखे हैं?	1. Yes<br>2. No	1. हाँ<br>2. नहीं	1<br>2	
7b	***If yes, in 7a ,*** what do you recall about these posters?<br><br>यदि हाँ, तो इन पोस्टरों के बारे में आपको क्या याद है?	a. Care during pregnancy<br>b. What to eat during pregnancy<br>c. Complementary feeding practices for children<br>d. Schemes related posters<br>e. Family responsibility<br>f. Others	a. गर्भावस्था के दौरान देखभाल<br>b. गर्भावस्था के दौरान क्या खाएं<br>c. बच्चों को पूरक आहार<br>d. योजनाओं से संबंधित पोस्टर<br>e. परिवार की जिम्मेदारी<br>f. अन्य कोई	a<br>b<br>c<br>d<br>e<br>f	
8a	Have you attended any VHSNC meeting (sitting in circle) conducted by ASHA in the last three months?<br><br>क्या आपने पिछले तीन महीनों में किसी ग्राम स्वास्थ्य, स्वच्छता एवं पोषण समिति की बैठक (आशा द्वारा गोल घेरे में बैठ के) में भाग लिया है	1. Yes<br>2. No	1. हाँ<br>2. नहीं	1<br>2	**If No, then go to Q 8F**
8b	***If yes in 8a***, how many meeting have you attended?<br><br>यदि हाँ, तो आपने कितनी बैठकों में भाग लिया है?	1. 3 or more<br>2. 3<br>3. 2<br>4. 1	1. 3 से ज़्यादा<br>2. 3<br>3. 2<br>4. 1	1<br>2<br>3<br>4	

CONFIDENTIAL

8c	***If yes in 8a***, Who conducted these meetings?<br><br>इन बैठकों का संचालन किसने किया?	1. ASHA<br>2. AWW<br>3. Other	1. आशा<br>2. आंगनवाड़ी कार्यकर्ता<br>3. अन्य कोई	1<br>2<br>3	
8d	***If yes in 8a***, Did you recall anything from these meeting/s?<br><br>क्या आपको इन मीटिंगों में से कुछ याद है?	1. Yes<br>2. No	1. हाँ<br>2. नहीं	1<br>2	
8e	If yes in 8d, what do you recall?<br><br>यदि हाँ, तो आपको क्या याद है?	a. Preparation before delivery- 'When Where Why' Games<br>b. Pregnancy related schemes such as IGMPY, JSY etc and available services<br>c. Three delays hindering safe delivery<br>d. Understanding food diversity and local foods and build an understanding on a balanced diet platter<br>e. Ways to make nutritious food and importance of consuming nutritious food for pregnant women,	a. प्रसव पूर्व तैयारी – 'कब कहां किस लिए' का खेल<br>b. गर्भावस्था सम्बंधित योजनाओं जैसे IGMPY, JSY आदि और उपलब्ध सेवाओं को जानना<br>c. सुरक्षित प्रसव में बाधक तीन देरियां<br>d. खाद्य विविधता और स्थानीय खाद्य पदार्थों को समझना और संतुलित आहार की थाली पर समझ बनाना<br>e. पौष्टिक आहार बनाने के तरीके एवं गर्भवती, धात्री, शिशु के लिए पौष्टिक आहार के सेवन का महत्व<br>f. गर्भावस्था और प्रसव के दौरान, प्रसव के बाद की जटिलताओं और देखभाल पर चर्चा - भंवरी की कहानी<br>g. अधिक जोखिम वाले बच्चे<br>h. जन्म के समय कम वजन वाले शिशुओं या जुड़वा बच्चों की देखभाल करना<br>i. जन्म के समय कम वजन वाले शिशुओं या जुड़वा बच्चों को स्तनपान कराना<br>j. छोटे बच्चों को गर्म रखने के कारण और प्रक्रिया	a<br>b<br>c<br>d<br>e<br>f<br>g<br>h<br>i<br>j<br>k<br>l<br>m<br>n<br>o<br>p<br>q<br>r<br>s<br>t<br>u<br>v	Additio n

CONFIDENTIAL

			lactating mothers and babies<br>f. Discussion on postpartum complications and care during pregnancy and delivery - Bhanwari's story<br>g. Babies most at risk<br>h. Dealing with low birth weight babies or twins<br>i. Breastfeeding of low birth weight babies or twins<br>j. Reasons and process of keeping young children warm<br>k. Breastfeed babies up to 6 months<br>l. Details of feeding the baby older than 6 months with supplementary food<br>m. *Annaprashan* Program	k. 6 माह तक के बच्चों को स्तनपान कराएं<br>l. 6 माह से अधिक उम्र के बच्चे को पूरक आहार खिलाने का विवरण<br>m. अन्नप्राशन कार्यक्रम<br>n. 6 महीने के बच्चों को खीर, दलिया आदि खिलाया गया<br>o. हाथ होने का तरीका सिखाया गया<br>p. ममता कार्ड पर चर्चा<br>q. चार्ट की मदद से वजन निगरानी करना<br>r. कुपोषित बच्चों की देखभाल करना<br>s. बच्चों को लगने वाले टीके पर चर्चा<br>t. दस्त पर चर्चा<br>u. दस्त के उपचार में ओआरएस और जिंक की गोली का उपयोग<br>v. दस्त की कहानी / सरिता और शबनम की कहानी<br>w. ओआरएस का घोल बनाना<br>x. हाथ धोने के बाद पानी को काँच के गिलास मे लेकर तुलना करना	w<br>x	

CONFIDENTIAL

		n. *Kheer, Daliya* etc. were fed to 6-month-old babies<br>o. Handwashing<br>p. Discussion on Mamta Card<br>q. Use of growth chart for weight monitoring<br>r. Care of a malnourished child<br>s. Child immunization<br>t. Discussion on diarrhea<br>u. Use of Zinc & ORS for the treatment of diarrhea<br>v. Diarrhea story / Story of Sarita & Shabnam<br>w. Preparation of ORS solution<br>x. Handwashing and comparison of waste water in glass			
8f	Have you attended any big community meeting conducted by ASHA in the last three months?	1. Yes<br>2. No	1. हाँ<br>2. नहीं	**If No, then**	

CONFIDENTIAL

	क्या आपने पिछले तीन महीनों में आशा द्वारा आयोजित किसी ग्राम स्वच्छता एवं पोषण समिति की बड़ी सामुदायिक बैठक में भाग लिया			**go to Q 8J**	
8g	**If yes in 8f**, Who conducted this meeting?<br><br>इस बैठक का संचालन किसने किया?	1. ASHA<br>2. AWW<br>3. Other	1. आशा<br>2. आंगनवाड़ी कार्यकर्ता<br>3. अन्य कोई	1<br>2<br>3	
8h	**If yes in 8f** ,Did you recall anything from these meeting/s?<br><br>क्या आपको इस मीटिंग में से कुछ याद है?	1. Yes<br>2. No	1. हाँ<br>2. नहीं	1<br>2	
8i	If yes in 8h, what do you recall?<br><br>यदि हाँ, तो आपको क्या याद है?	**Discussion on lessons learned from the first 4 meetings**<br>a. Power of unity – Breaking the sticks.<br>b. Identification of persons who have been denied access to most services - Kadam ka Khel<br>c. Identifying village level issues related to nutrition- Pebble Game<br>d. Strategies to solve identified problems<br>**e.** Community involvement to improve nutrition and health of mother and child	**पहली 4 बैठकों की सीख पर चर्चा**<br>a. संगठन में शक्ति हैं - लकड़ी तोड़ने वाला खेल<br>b. उन व्यक्तियों की पहचान जो अधिकतर सेवाओं तक पहुंच से वंचित रह गए हैं - कदम का खेल<br>c. पोषण से संबंधित ग्राम स्तर के मुद्दों की पहचान करना; मुख्य रूप से कुपोषण - चुनाव का खेल<br>d. पहचानी गई समस्याओं के समाधान की रणनीति<br>e. मां और शिशु के पोषण और स्वास्थ्य में सुधार लाने के लिए समुदाय की भागीदारी	a<br>b<br>c<br>d<br>e	
8j	How many VHSNC meetings (sitting in circle) conducted by ASHA have you attended till now?<br><br>अब तक आपने आशा द्वारा आयोजित वीएचएसएनसी की कितनी बैठकें (घेरे में बैठकर) की हैं?	.....(Number) **[RANGE 0-24 times]**	.... (संख्या)) **[RANGE 0-24 times]**		This question is currently conditioned under 8f. It will be independent

CONFIDENTIAL

					of that. The question will appear after 8f if saying NO in 8f.
	**IPC exposure**				
9a	In the last three months did the Poshan Champion (PC) visit your household?<br><br>पिछले तीन महीनों में पोषण चैंपियन (PC) ने आपके घर का दौरा किया?	1. Yes<br>2. No	1. हाँ<br>2. नहीं	1<br>2	
9b	***If yes, in 9a,*** how many times?<br>यदि हाँ, तो कितनी बार?	1. 3<br>2. 2<br>3. 1	1. 3<br>2. 2<br>3. 1	3<br>2<br>1	
9c	During the conversation did She/ He use any job aid?<br><br>बातचीत के दौरान, उन्होंने किसी Job Aid का उपयोग किया था?	1. Yes<br>2. No	1. हाँ<br>2. नहीं	1<br>2	
9d	***If yes in 9c,*** what among these were used during the conversation?<br><br>यदि हाँ, तो बातचीत के दौरान इनमें से क्या इस्तेमाल किया गया था?	a. Phone app with videos<br>b. Flip book (Job Aid)<br>c. Any other<br>d. Nothing	a. फोन ऐप वीडियो के साथ<br>b. फ्लिप बुक (जॉब ऐड)<br>c. अन्य कोई<br>d. कुछ नहीं	a<br>b<br>c<br>d<br>e	
9e	Did you find this conversation helpful?<br><br>क्या आपको ये बातचीत मददगार लगी ?	1. Yes<br>2. No<br>3. Can't say	1. हाँ<br>2. नहीं<br>3. कह नहीं सकते	1<br>2<br>3	
10a	In the last three months did ASHA visit your household?<br><br>पिछले तीन महीनों में, आशा ने आपके घर का दौरा किया?	1. Yes<br>2. No	1. हाँ<br>2. नहीं	1<br>2	
10b	If yes how many times?<br><br>यदि हाँ, तो कितनी बार?	Number<br>**(RANGE 0-90 days)**	Number		

CONFIDENTIAL

11a	In the last three months did AWW visit your household?<br>पिछले तीन महीनों में, AWW ने आपके घर का दौरा किया?	1. Yes<br>2. No	1. हाँ<br>2. नहीं	1<br>2	
11b	If yes how many times?<br><br>यदि हाँ तो कितनी बार?	Number<br>**(RANGE 0-90 days)**	Number		
12a	In the last three months you met ANM visit your household or you met her?<br><br>पिछले तीन महीनों में, ANM ने आपके घर का दौरा किया?	1. Yes<br>2. No	1. हाँ<br>2. नहीं	1<br>2	
12b	If yes how many times?<br><br>यदि हाँ, तो कितनी बार?	Number<br>**(RANGE 0-90 days)**	Number		
13	***If yes in any of the 9a 10a 11a or 12a***; what you can recall which they do OR tell you during their visit?<br>**DO NOT PROMPT**	In the below option we need to give conditionality as indicated below			
	If pregnancy stage was (PW 1 to PW4 only)<br>A. Importance of weight gain during pregnancy<br>B. How much weight a PW should gain<br>C. How many times a PW should eat<br>D. What to eat during pregnancy<br>E. How to take IFA and calcium tables<br>F. When to take IFA and calcium tables<br>G. Advised 2 hours of daytime rest<br>H. Get every month weight measurement<br>I. Get regular ANC check-up<br>J. Discussed about PMMVY/IGMPY<br>K. About the treatment and prevention of anemia<br><br>***If pregnancy stage was (PW 3 and PW4 only)***<br>L. Preparedness for safe birth (hospital ambulance keep Mamta card)	***यदि गर्भावस्था अवस्था थी (PW 1 to PW4 only)***<br>a. गर्भावस्था के दौरान वजन बढ़ने का महत्व<br>b. गर्भवती महिला का कितना वजन बढ़ना चाहिए<br>c. PW को कितनी बार खाना चाहिए<br>d. गर्भावस्था के दौरान क्या खाएं<br>e. IFA और कैल्शियम की गोलियाँ कैसे लेने के लिए<br>f. IFA और कैल्शियम की गोलियां कब लेनी हैं<br>g. दिन मे 2 घंटे आराम करने की सलाह दी<br>h. हर महीने वजन माप लें<br>i. नियमित एएनसी जांच करवाएं<br>j. PMMVY / IGMPY के बारे में चर्चा की<br>k. एनीमिया के उपचार और रोकथाम के बारे में<br><br>***यदि गर्भावस्था अवस्था (PW3 to PW4 only)***	***If Lactating mother (LM 1 - 3.5 month)***<br>a. Child weight/height gain as per age (growth monitoring)<br>b. Child development as per stage<br>c. Cord care<br>d. KMC<br>e. Exclusive breastfeeding (in first 6 months only mother milk and not even water)<br>f. Child timely vaccination as per age<br>g. Mother's nutrition- What to eat during lactation	***यदि स्तनपान कराने वाली मां (LM 1 - 3.5 month)***<br>a. आयु के अनुसार बच्चे का वजन / ऊँचाई बढ़ना (विकास निगरानी)<br>b. स्टेज के अनुसार बच्चे का विकास<br>c. गर्भनाल की देखभाल<br>d. कंगारू मदर केयर (मां के सीने से बच्चे को चिपकाना)<br>e. केवल स्तनपान (पहले 6 महीनों में केवल माँ का दूध और पानी भी नहीं)<br>f. उम्र के अनुसार बच्चे का समय पर टीकाकरण<br>g. माँ का पोषण- स्तनपान के दौरान क्या खाएं<br>h. PMMVY / IGMPY के बारे में चर्चा की	

CONFIDENTIAL

	M. Breastfeeding within one hour<br>N. Kangaroo Mother Care<br>O. Cord care (nothing on cord)<br>P. Exclusive breastfeeding (in first 6 months/ no water)<br>Q. About Family Planning<br>R. About the treatment and prevention of anemia	l. सुरक्षित जन्म के लिए तैयारी (अस्पताल, एम्बुलेंस, ममता कार्ड रखें)<br>m. एक घंटे के भीतर स्तनपान<br>n. कंगारू मदर केयर<br>o. गर्भनाल देखभाल (गर्भनाल पर कुछ नहीं)<br>p. केवल स्तनपान स्तनपान (पहले 6 महीने / पानी नहीं)<br>q. परिवार नियोजन के बारे में<br>r. एनीमिया के उपचार और रोकथाम के बारे में	h. Discussed about PMMVY/IGMPY<br>i. About Family Planning<br><br>***<u>If Mother of Young child (MY)</u>***<br>a. Measured child's MUAC<br>b. Measured child's height<br>c. Measured child's weight<br>d. What to feed to the child after 6 months<br>e. About Family Planning	i. परिवार नियोजन के बारे में<br><br>***<u>यदि युवा बच्चे की माँ (MY)</u>***<br>a. बच्चे का MUAC मापा गया<br>b. बच्चे की ऊंचाई की माप<br>c. बच्चे के वजन की माप<br>d. 6 महीने के बाद बच्चे को क्या खिलाना है<br>e. परिवार नियोजन के बारे में	
14a	On 9th of the last month did you visit PHC/CHC last month?<br>पिछले महीने की 9 तारीख को, आपने पीएचसी / सीएचसी का दौरा किया था?	1. Yes<br>2. No	1. हाँ<br>2. नहीं	1<br>2	
14b	Were you referred by ANM to visit PHC/CHC during last month?<br>क्या आपको एएनएम ने पिछले महीने पीएचसी/सीएचसी में जाने के लिए रेफर किया था?	1. Yes<br>2. No	1. हाँ<br>2. नहीं		
14c	Were you counselled?<br><br>क्या आपको काउंसलिंग मिली थी?	1. Yes<br>2. No	1. हाँ<br>2. नहीं	1<br>2	
14d	What you recall from that?<br><br>उससे आपको क्या याद है?	a. The need for ante-natal care (ANC) checkups for women<br>b. Consumption of one IFA tablet a day<br>c. During pregnancy Eating nutritive diet and healthy food (What to eat and what not to eat)<br>d. Importance of weight gain during pregnancy<br>e. How much weight a PW should gain<br>f. Breastfeeding within one hour	a. महिलाओं के लिए प्रसव पूर्व देखभाल (एएनसी) चेकअप की आवश्यकता<br>b. गर्भावस्था के दौरान एक दिन में एक IFA टैबलेट का सेवन<br>c. पौष्टिक आहार / स्वस्थ भोजन खाना (क्या खाना चाहिए और क्या नहीं खाना चाहिए)<br>d. गर्भावस्था के दौरान वजन बढ़ने का महत्व<br>e. PW का कितना वजन बढ़ना चाहिए<br>f. एक घंटे के भीतर स्तनपान	a<br>b<br>c<br>d<br>e<br>f<br>g	

CONFIDENTIAL

		g. Exclusive breastfeeding (in first 6 months only mother milk and not even water)<br>h. Importance of regular growth monitoring<br>i. Anemia prevention and treatment	g. केवल स्तनपान (पहले 6 महीनों में केवल माँ का दूध और पानी भी नहीं)<br>h. वजन निगरानी का महत्व<br>i. एनीमिया के उपचार और रोकथाम के बारे में	h	
15a	Have you participated in Suposhan Diwas during last 3 months?<br><br>क्या आपने पिछले 3 महीने के दौरान सुपोषण दिवस में भाग लिया है?	1. Yes<br>2. No	1. हाँ<br>2. नहीं	1<br>2	
15b	If yes, what you recall from the Suposhan Diwas meeting?<br><br>आप सुपोषण दिवस बैठक से क्या याद हैं?	a. The need for ante-natal care (ANC) checkups for women<br>b. Consumption of one IFA tablet a day during pregnancy Eating nutritive diet and healthy food<br>c. Importance of weight gain during pregnancy<br>d. How much weight a PW should gain<br>e. Breastfeeding within one hour<br>f. Exclusive breastfeeding (in first 6 months only mother milk and not even water)<br>g. Importance of regular growth monitoring<br>h. Importance of complementary feeding	a. महिलाओं के लिए प्रसव पूर्व देखभाल (एएनसी) चेकअप की आवश्यकता<br>b. गर्भावस्था के दौरान एक दिन में एक IFAटैबलेट का सेवन पौष्टिक आहार / स्वस्थ भोजन खाना<br>c. गर्भावस्था के दौरान वजन बढ़ने का महत्व<br>d. PW का कितना वजन बढ़ना चाहिए<br>e. एक घंटे के भीतर स्तनपान<br>f. केवल स्तनपान (पहले 6 महीनों में केवल माँ का दूध और पानी भी नहीं)<br>g. वजन निगरानी का महत्व<br>h. पूरक भोजन की मात्रा और उत्तरदायी भोजन		
15c	***If PW 3 AND PW4 only:***<br>Have you participated in Garbhavastha Paramarsh Diwas/Godhbharai during last month?<br><br>क्या आपने पिछले 3 महीने के दौरान गर्भवस्था परामर्श /गोधभराई दिवस में भाग लिया है?	1. Yes<br>2. No	1. हाँ<br>2. नहीं		

CONFIDENTIAL

15d	If yes, what you recall from the Garbhavastha Paramarsh Diwas/Godhbharai meeting?<br><br>गर्भवस्था परामर्श /गोधभराई दिवस बैठक से क्या याद हैं?	a. Benefits of Cash transfer schemes (PMMVY/IGMPY/JSY/RajShree)<br>b. Importance of Mamta card<br>c. The need for ante-natal care (ANC) checkups for women<br>d. Importance of maternal nutrition	a) नकद हस्तांतरण योजनाओं के लाभ (पीएमएमवीवाई/आईजीएमपीवाई/जेएसवाई/ राजश्री)<br>b) ममता कार्ड का महत्व<br>c) महिलाओं के लिए प्रसवपूर्व देखभाल (एएनसी) जांच की आवश्यकता<br>d) मातृ पोषण का महत्व		
15e	From mother of 6-12 months children: Have you participated in Annaprashan diwas during last 3 months?<br><br>क्या आपने पिछले 3 महीने के दौरान अन्नप्राशन दिवस में भाग लिया है?	1. Yes<br>2. No	1. हाँ<br>2. नहीं		
15f	If yes, what you recall from the Annaprashan diwas meeting?<br><br>अन्नप्राशन दिवस बैठक से क्या याद हैं?	a. Importance of complementary feeding<br>b. Importance of regular growth monitoring<br>c. Continuation of breast feeding	a. पूरक भोजन की मात्रा और उत्तरदायी भोजन<br>b. वजन निगरानी का महत्व<br>c. स्तनपान जारी रखना		
16a	Have you attended MCHND meeting whrere services are provided by ANM in last three months?<br><br>आप पिछले 3 महीने में ANM द्वारा Mamta Divas/ Vaccination Day/MCHND बैठकों में भाग लेने गए थे?	1. Yes<br>2. No	1. हाँ<br>2. नहीं	1<br>2	
16b	If yes how many meetings have you attended?<br><br>अगर हां, तो कितनी बार आप Mamta Divas/ Vaccination Day/MCHND बैठकों में भाग लिया है?	Number<br>**(RANGE 0-6 times)**	Number		

CONFIDENTIAL

Section 5: Knowledge and Awareness and Practice (Respondents - PW LM MY)		Options in English	Options in Hindi	Code	Remark
1	What are the things to remember during pregnancy?<br><br>गर्भावस्था के दौरान किन चीजों को याद रखना चाहिए	a. Eat frequent and more food<br>b. Eat nutritious food with 5 nutrients<br>c. Eat additional snacks<br>d. Daily intake of IFA and calcium tablets<br>e. Every month weight measurement<br>f. Rest for 2 hours in day time	a. अधिक आवृत्ति के साथ अधिक भोजन करें<br>b. 5 पोषक तत्वों के साथ पौष्टिक आहार लें<br>c. अतिरिक्त स्नैक्स खाएं<br>d. IFA (Iron) और कैल्शियम तालिकाओं का दैनिक सेवन<br>e. हर महीने वजन माप<br>f. दिन के समय में 2 घंटे आराम करें	a<br>b<br>c<br>d<br>e<br>f	Multiple choice
2a	During pregnancy how many times should a pregnant woman take her meals<br><br>गर्भावस्था के दौरान, गर्भवती महिला को कितनी बार भोजन लेना चाहिए?	No. of times - Meals	संख्या - भोजन		
2b	What types of nutritious food a pregnant woman should eat during her pregnancy?<br><br>गर्भावस्था के दौरान गर्भवती महिला को किस प्रकार के पौष्टिक भोजन खाने चाहिए?	a. Daily Green vegetables<br>b. Daily Dal<br>c. Daily fresh fruit<br>d. Daily Milk or any milk products<br>e. Eggs or meat if non-veg<br>f. Gur chana<br>Moongphali (nuts and seeds)	a. हरी सब्जियाँ<br>b. दाल<br>c. ताजा फल<br>d. दूध या कोई भी दुग्ध उत्पाद<br>e. अंडा या मांस (यदि मांसाहारी हो)<br>f. गुर चना<br>मूंगफली (नट और बीज)	Yes No<br>हाँ नहीं<br>1 2<br>1 2<br>1 2<br>1 2<br>1 2<br>1 2	
3a	During pregnancy other than regular meals how many times should a pregnant woman take her snacks?<br><br>गर्भावस्था के दौरान, नियमित भोजन के अलावा, गर्भवती महिला को कितनी बार नाश्ता लेना चाहिए?	No. of times - Snacks -	संख्या - नाश्ता		
3b	What as additional snacks a pregnant woman should eat during her pregnancy?<br><br>***Unprompted***	a) Local fruit /juice<br>b) Sweet potato<br>c) Milk and milk products<br>d) Rabadi<br>e) Raab/ Rabadi<br>f) Lassi/ Chhach<br>g) Gur and Moongfali	a. स्थानीय फल/फलों का रस<br>b. शकरकंद<br>c. दूध या दूध से बने पदार्थ / दही<br>d. रबाड़ी<br>e. राब / रबाड़ी<br>f. लस्सी / छाछ	a<br>b<br>c<br>d<br>e<br>f<br>g	Multiple choice

CONFIDENTIAL

	एक गर्भवती महिला को गर्भावस्था के दौरान अतिरिक्त स्नैक्स के रूप में क्या खाना चाहिए?	h) Peanuts<br>i) Chikki<br>j) Chiwada/Murmura/Bhelpuri<br>k) Halwa<br>l) Ladoo<br>m) Upma<br>n) Poha<br>o) Daliya<br>p) Biscuit<br>q) Samosa/ Pokada<br>r) Matri<br>s) Other- Maggie, Kurkure/Tanatan/cold drinks/ Icecream/soda/ etc<br>t) Don't know	g. गुड़ मूंगफली<br>h. मूंगफली<br>i. चिकी<br>j. चिवड़ा / मुरमुरा / भेलपुरी<br>k. हलवा (मूंगदाल, बेसन, सूजी, आटा या बहु अनाज)<br>l. लड्डू -(तिल, बेसन, सूजी, आटा या बहु अनाज)<br>m. उपमा<br>n. पोहा<br>o. दलिया<br>p. बिस्कुट<br>q. समोसा/पकोड़ा<br>r. मठरी<br>s. अन्य- मैगी, कुरकुरे/टानाटन/कोल्ड ड्रिंक्स/आइसक्रीम/सोडा)<br>t. पता नहीं	h<br>i<br>j<br>k<br>l<br>m<br>n<br>o<br>p<br>q<br>r	
5	How many times should a pregnant woman undergo for ANC checkups during pregnancy?<br><br>एक गर्भवती महिला को अपनी पूरी गर्भावस्था के दौरान कितनी बार ANC चेकअप कराना चाहिए?	Number<br>0<br>1<br>2<br>3<br>4<br>5<br>6<br>7 Don't know	संख्या<br>0<br>1<br>2<br>3<br>4<br>5<br>6<br>7 पता नहीं	1<br>2<br>3<br>4<br>5<br>6<br>7	
6	How much weight should a pregnant woman gain during pregnancy period?<br>एक गर्भवती महिला को अपनी गर्भावस्था की अवधि के दौरान कितना वजन हासिल करना चाहिए?	1. >12 Kg<br>**2. 10-12 Kg**<br>3. 8-10 Kg<br>4. 6-8 Kg<br>5. <6 Kg<br>6. Don't know	1. >12 Kg<br>2. 10-12 Kg<br>3. 8-10 Kg<br>4. 6-8 Kg<br>5. <6 Kg<br>6. पता नहीं	1<br>2<br>3<br>4<br>5<br>6	Single choice

CONFIDENTIAL

7	After the 4th month how much weight should a pregnant woman gain per month?<br>चौथे महीने के बाद गर्भवती महिला को हर महीने कितना वजन बढ़ना चाहिए?	1. < 1 Kg<br>2. 1 Kg to 1.5 Kg<br>3. 1.5 to 2 Kg<br>4. > 2 Kg<br>5. Don't know	1. < 1 Kg<br>2. 1 Kg to 1.5 Kg<br>3. 1.5 to 2 Kg<br>4. > 2 Kg<br>5. पता नहीं	1<br>2<br>3<br>4<br>5	Single choice
8	With what a pregnant woman consume IFA tablets?<br><br>गर्भवती महिला को आइएफए गोलियों का सेवन किसके साथ करना चाहिए?	1. With water<br>2. With lemon water<br>3. With milk<br>4. With juice	1. पानी के साथ<br>2. नींबू पानी के साथ<br>3. दूध के साथ<br>4. रस के साथ	1<br>2<br>3<br>4	Single choice
9	With what a pregnant woman consume Calcium tablets?<br><br>गर्भवती महिला को कैल्शियम की गोलियों का सेवन किसके साथ करना चाहिए?	1. With water<br>2. With lemon water<br>3. With milk<br>4. With juice	1. पानी के साथ<br>2. नींबू पानी के साथ<br>3. दूध के साथ<br>4. रस के साथ	1<br>2<br>3<br>4	
10	Can the IFA & Calcium tablets taken together?<br>क्या IFA (Iron) और Calcium की गोलियाँ एक साथ ली जा सकती हैं?	1. Yes<br>2. No	1. हाँ<br>2. नहीं	1<br>2	
11	***If No in 10*** what should be the minimum interval between consuming IFA & Calcium tablets?<br><br>यदि नहीं, तो IFA के उपभोग के बीच न्यूनतम अंतराल क्या होना चाहिए	1. < 4 hours<br>2. > 4 hours<br>3. Don't Know	1.<4 घंटे<br>2. >4 घंटे<br>3. पता नहीं	1<br>2<br>3	Single choice
12	What are other source of iron in food?<br><br>निम्नलिखित में से भोजन में आयरन के अन्य स्रोत क्या हैं?	a. Green leafy vegetables<br>b. Jaggary<br>c. Don't Know	a. "गहरा हरे पत्ते वाली सब्जियां<br>b. गुड<br>c. पता नहीं	a<br>b<br>c	
13	What are other source of calcium in food?<br><br>निम्नलिखित में से भोजन में कैल्शियम के अन्य स्रोत क्या हैं?	a. Milk and milk products<br>b. Fruits<br>c. Don't Know	a. दूध और दूध उत्पाद<br>b. फल<br>c. पता नहीं	a<br>b<br>c	

CONFIDENTIAL

14	What should a baby be fed immediately after birth?<br><br>जन्म के तुरंत बाद बच्चे को क्या खिलाना चाहिए?	1. Water<br>2. Honey/ Jaggary<br>3. Homemade syrup/fluids<br>4. Cow (animal) Milk<br>5. Janm Ghutti<br>6. Formula Milk for babies<br>7. Only colostrum/ Mother's Milk<br>8. Don't Know	1. पानी<br>2. शहद / गुड़<br>3. घर का बना सिरप / तरल पदार्थ<br>4. गाय (पशु) दूध<br>5. जनम घुमती<br>6. शिशुओं के लिए फार्मूला मिल्क<br>7. केवल कोलोस्ट्रम / मदर मिल्क<br>8. पता नहीं	1<br>2<br>3<br>4<br>5<br>6<br>7<br>8	Single choice
15	When should a newborn be put to the breast for the first time after birth?<br><br>जन्म के बाद पहली बार एक नवजात शिशु को कब स्तन पान करना चाहिए?	1. Immediately after birth within 30 minutes<br>2. Within the first hour after delivery<br>3. Within 24 hours<br>4. A day or more after delivery<br>5. Don't Know	1. जन्म के तुरंत बाद 30 मिनट के भीतर<br>2. प्रसव के बाद पहले घंटे के भीतर<br>3. चौबीस घंटों के भीतर<br>4. प्रसव के बाद एक दिन या उससे अधिक<br>5. पता नहीं	1<br>2<br>3<br>4<br>5	Single choice
16	For how long does a child need to be given ONLY breast-milk/mother's milk and nothing else (not even water)?<br><br>एक बच्चे को केवल स्तन-दूध / माँ का दूध कब तक देने की आवश्यकता है और कुछ नहीं (पानी भी नहीं)?	Number of months	महीनों की संख्या		
17	By which month should we start giving water to a child?<br><br>किस महीने से, हमें बच्चे को पानी देना शुरू कर देना चाहिए?	Number	संख्या		
18	At what age (month) a child should be introduced to Complementary (Semi-solid or soft food<br>) food?<br><br>किस उम्र (महीने) में एक बच्चे को मानार्थ भोजन देना चाहिए?	Number	संख्या		
19	After 6 months what should be given as complimentary to child?	1. Semi-solid or soft food<br><br>2. Other response	1. अर्ध-ठोस या नरम भोजन<br><br>2. अन्य प्रतिक्रिया	1<br><br>2	

CONFIDENTIAL

	6 महीने के बाद, बच्चे को पूरक के रूप में क्या दिया जाना चाहिए?				
20	How many times **a 6-8 months** child should be given complementary semi-solid or soft food in a day?<br><br>6-8 महीने के बच्चे को कितनी बार पूरक अर्ध-ठोस या नरम भोजन दिया जाना	1. 2-3 meals a day<br>2. 3-4 meals a day<br>3. DK	1. दिन में 2-3 बार भोजन<br>2. दिन में 3-4 बार भोजन<br>3. DK	1<br>2<br>3	

CONFIDENTIAL

21	*Myth in community; Read one by one and ask - Change the heading and please keep only 4 questions which are given below remove all other questions)*<br>**As per your opinion tell me about the following…………… is this true or false?**<br>***मिथक आधारित प्रश्न; एक एक करके पढ़ें और पूछें***<br>आपकी राय के अनुसार, मुझे निम्नलिखित के बारे में बताएं - सही या गलत?	**Respondents - PW LM MY**
A	Eating banana during pregnancy can stick foetus to the uterus	True - 1 False – 2 DK - 3
	गर्भावस्था के दौरान केला खाने से भ्रूण गर्भाशय से चिपक सकता है	सही -1 गलत – 2 पता नहीं-3
B	Milk taken during pregnancy can stick to the body of the foetus which also causes white spots	True - 1 False – 2 DK - 3
	गर्भावस्था के दौरान लिया गया दूध गर्भस्थ शिशु के शरीर में चिपक सकता है, जिससे सफेद धब्बे भी होते हैं	सही -1 गलत – 2 पता नहीं-3
C	Ghutti given to a newborn is useful	True - 1 False – 2 DK - 3
	नवजात को दी जाने वाली घुट्टी उपयोगी होती है	सही -1 गलत – 2 पता नहीं-3
D	Eating jaggery during pregnancy produces body heat which is harmful for pregnancy	True - 1 False – 2 DK - 3
	गर्भावस्था के दौरान गुड़ खाने से शरीर में गर्मी पैदा होती है जो गर्भावस्था के लिए हानिकारक है।	सही -1 गलत – 2 पता नहीं-3

**Section 6: Receipt and Use of Cash by PW/LM/ MY**		**Options in English**	**Options in Hindi**	**Code**	**Remark**
1a	***Ask if first time pregnant or have first child (PMMVY eligible women only)***<br>*पूछें कि क्या पहली बार गर्भवती हैं या पहला बच्चा है*<br>Are you registered in PMMVY cash transfer Scheme?<br>क्या आप पीएमएमवीवाई नकद हस्तांतरण योजना में पंजीकृत हैं?	1. Yes<br>2. No<br>3. Don't know	1. हाँ<br>2. नहीं<br>3. पता नहीं	1<br>2<br>3	
1b	If yes, did you receive installment of PMMVY cash payment?<br>यदि हाँ, तो क्या आपको PMMVY नकद भुगतान की किस्त मिली है?	1. Yes<br>2. No<br>3. Don't know	1. हाँ<br>2. नहीं<br>3. पता नहीं	1<br>2<br>3	
1c	If yes, how many instalments of cash payment you have received so far?<br>यदि हाँ, तो अब तक आपको नकद भुगतान की कितनी किश्तें प्राप्त हुई हैं?	No. of Instalments	किस्तों की संख्या		

CONFIDENTIAL

2a	***Ask if Second time pregnant or second child <u>(IGMPY eligible women only)</u>***<br>*पूछे की क्या वो दूसरी बार की गर्भवती है अथवा 2 जीवित बच्चे है*<br>Are you registered in IGMPY cash transfer Scheme?<br>क्या आप IGMPY नकद हस्तांतरण योजना में पंजीकृत हैं?	1. Yes<br>2. No<br>3. Don't know	1. हाँ<br>2. नहीं<br>3. पता नहीं	1<br>2<br>3	
2b	If yes did you receive installment of IGMPY cash payment?<br>यदि हाँ, तो क्या आपने IGMPY नकद भुगतान की किस्त प्राप्त की है?	1. Yes<br>2. No<br>3. Don't know	1. हाँ<br>2. नहीं<br>3. पता नहीं	1<br>2<br>3	
2c	If yes how much cash amount have you received so far?<br>यदि हाँ, तो आपको अब तक कितनी किश्तें मिली हैं?	1. No. of Instalments<br>2. Don't Know	1. किस्तों की संख्या<br>2. पता नहीं	1<br>2	
3a	If yes how much cash you have withdrawn so far?<br>यदि हाँ, तो आपने अब तक कितनी नकदी निकाली है?	|__|__|__|__|	|__|__|__|__|	|__|__|__|__|	
3b	Who access your bank account for withdrawal?<br>आपके बैंक खाते में से पैसे निकलवाने के लिए कौन जाता है?	1. Me<br>2. My husband<br>3. Other<br>4. Don't Know	1. मैं स्वयं<br>2. मेरे पति<br>3. अन्य<br>4. पता नहीं	1<br>2<br>3<br>4	
3c	If received for what purpose did you mainly use this money?<br>यदि पैसा प्राप्त किया गया है, तो आप किस उद्देश्य के लिए मुख्य रूप से इस पैसे का उपयोग करते हैं?	A. Bought food for my own consumption<br>B. Bought food for the entire household<br>C. Bought animal for milk<br>D. Paid for health care expenditure of family<br>E. Paid for immediate household needs (such as clothes small items)<br>F. Paid child's school/ tuition fees<br>G. Paid for other needs (loan house repair etc.)<br>H. Not yet used any installment	A. मेरे स्वयं के उपभोग के लिए भोजन खरीदा<br>B. पूरे घर का खाना खरीदा<br>C. दूध के लिए जानवर खरीदे<br>D. परिवार के स्वास्थ्य देखभाल खर्च के लिए भुगतान किया<br>E. घरेलू जरूरतों के लिए भुगतान (जैसे कपड़े, छोटी वस्तुएं)<br>F. बच्चों के स्कूल / ट्यूशन फीस का भुगतान किया<br>G. अन्य जरूरतों के लिए भुगतान (ऋण, घर की मरम्मत, आदि)<br>H. अभी तक किसी भी किस्त का इस्तेमाल नहीं किया	Yes No<br>हाँ नहीं<br>1 2<br>1 2<br>1 2<br>1 2<br>1 2<br>1 2<br>1 2<br>1 2<br>1 2	Multiple choice

CONFIDENTIAL

4	If no to Q1b/Q2b why did you not receive the cash?<br><br>यदि Q1b / Q2b के लिए नहीं, तो आपको नकद क्यों नहीं मिला?	1. Documents incomplete for submission<br>2. Received the benefit earlier<br>3. Do not know<br>4. Other Specify ____	1. प्रस्तुत करने के लिए दस्तावेज अपूर्ण थे<br>2. पूर्व में लाभ प्राप्त कर चुके है<br>3. पता नहीं<br>4. अन्य (निर्दिष्ट करे)	1<br>2<br>3<br>4	
5.	Cash use for food during pregnancy and lactation period irrespective of schemes money received<br>योजना के पैसे प्राप्त करने के बावजूद, गर्भावस्था और स्तनपान अवधि के दौरान भोजन के लिए नकद उपयोग				
5.1	From Pregnant Women(PW)<br>Did you spend more money for your food during your pregnancy than normal time?<br><br>गर्भवती महिलाओं से (पीडब्ल्यू)<br>क्या आपने अपनी गर्भावस्था के दौरान सामान्य समय की तुलना में अपने भोजन पर अधिक पैसा खर्च किया?	1. Yes<br>2. No	1. हाँ<br>2. नहीं	1<br>2	
5.2	From Pregnant Women (PW)<br>If yes to Q5.1, how much more amount (Rs.) you spend for your food in a month during pregnancy?<br><br>गर्भवती महिलाओं से (पीडब्ल्यू)<br>यदि Q5.1 में हाँ, आप गर्भावस्था के दौरान एक महीने में अपने भोजन पर कितनी अधिक राशि (रु.) खर्च करती हैं?	....<br>(Amount in Indian Rupees)<br><br>**(RANGE Rs.1-Rs 9999)**	.... (राशि भारतीय रुपये में)	In values	
5.3	From Lactating Mother (LM)/ Mother of Young Child (MY)<br>Did you spend more money for your food during your lactation period than normal time?<br><br>स्तनपान कराने वाली माँ (LM)/ छोटे बच्चे की माँ (MY) से<br>क्या आपने अपने स्तनपान की अवधि के दौरान सामान्य समय की तुलना में अपने भोजन के लिए अधिक पैसा खर्च किया?	1. Yes<br>2. No	1. हाँ<br>2. नहीं	1<br>2	
5.4	From Lactating Mother (LM)/ Mother of Young Child (MY)<br>If yes to Q5.3, how much more amount (Rs.) you spend for your food in a month during your lactation period?<br>स्तनपान कराने वाली माँ (LM)/ छोटे बच्चे की माँ (MY) से	....<br>(Amount in Indian Rupees)	.... (राशि भारतीय रुपये में)	In values	

CONFIDENTIAL

	यदि Q5.3 में हाँ, आप अपने स्तनपान की अवधि के दौरान एक महीने में अपने भोजन के लिए कितनी अधिक राशि (रु.) खर्च करती हैं?	**(RANGE Rs.1-Rs 9999)**			
5.5	During your pregnancy and lactation, did you consume anything extra in addition to what you consume in regular meal?<br><br>(अपनी गर्भावस्था और स्तनपान के दौरान, क्या आपने नियमित भोजन के अलावा कुछ अतिरिक्त खाया है?)	1.Yes<br>2. No	1. हाँ<br>2. नहीं	1<br>2	

CONFIDENTIAL
